# Microfluidic techniques for enhancing biofuel and biorefinery industry based on microalgae

**DOI:** 10.1186/s13068-019-1369-z

**Published:** 2019-02-15

**Authors:** Pierre Bodénès, Hsiang-Yu Wang, Tsung-Hua Lee, Hung-Yu Chen, Chun-Yen Wang

**Affiliations:** 1 Department of Power Mechanical Engineering, National Tsing Hua University, Hsinchu, Taiwan; 2 Department of Engineering and System Science, National Tsing Hua University, Hsinchu, Taiwan; 3 Institute of Nuclear Science, National Tsing Hua University, Hsinchu, Taiwan; 4 Department of Chemical Engineering, National Cheng Kung University, Tainan, Taiwan

**Keywords:** Microfluidics, Microalgae, Screening, Metabolites production, Downstream treatments

## Abstract

This review presents a critical assessment of emerging microfluidic technologies for the application on biological productions of biofuels and other chemicals from microalgae. Comparisons of cell culture designs for the screening of microalgae strains and growth conditions are provided with three categories: mechanical traps, droplets, or microchambers. Emerging technologies for the in situ characterization of microalgae features and metabolites are also presented and evaluated. Biomass and secondary metabolite productivities obtained at microscale are compared with the values obtained at bulk scale to assess the feasibility of optimizing large-scale operations using microfluidic platforms. The recent studies in microsystems for microalgae pretreatment, fractionation and extraction of metabolites are also reviewed. Finally, comments toward future developments (high-pressure/-temperature process; solvent-resistant devices; omics analysis, including genome/epigenome, proteome, and metabolome; biofilm reactors) of microfluidic techniques for microalgae applications are provided.

## Background

Microalgae are considered as bio-based cell factories, able to rapidly colonize a liquid medium and produce a large variety of chemicals synthesized from their environment [1]. After biomass fractionation and purification processes, most of the chemicals can be valorized: intracellular lipids (transesterification to biodiesel, unsaturated fatty acids for healthy food), starch (fermentation to ethanol), chlorophyll, carotenoids, or phycobiliproteins pigments (feed, food, medical applications, cosmetics) [2]. Numerous efforts have been paid to finding prolific strains, enhancing biomass production, and shifting metabolic pathways to increase the yield of these products [3]. Bioreactor designs [4], microalgae harvest techniques [5], metabolite extraction methods [6], and downstream chemical/physical treatments [7] are also intensively studied to reduce the production costs. Nonetheless, the commercial production of many microalgal products still face the challenges of high production costs and low yields due to the low throughput and the high expense of using laboratory-scale or pilot-scale processes for optimizing the production. Microfluidic techniques have proven their high throughput and low cost in a number of microbial applications such as screening and directed evolution of prolific yeast strains [8, 9], detection of pathogenic microorganisms [10], and miniature microbial fuel cells [11]. Taking advantages of microfluidic techniques, the expediting of enhancement of microalgal fuel and the biorefinery industry is anticipated.

In a bio-based industry with concern, aiming to reach a high productivity in a specific high value product, one should select the most prolific microalgae species and the tailored conditions to maximize the production of targeted chemicals. To obtain the optimal strains and conditions, the first studies of microfluidic techniques for microalgae aimed to identify the characteristics of different strains and establish microscale bioreactors. Various microfluidic screening platforms have been designed to cultivate microalgae and study their growth at microscale [12]. Miniaturized systems are extremely convenient to monitor, in situ and on single cells, the impacts of culture conditions on microalgae morphology, viability, and accumulation of secondary metabolites such as lipid or pigments. Furthermore, culture conditions can be precisely mastered regarding fluidic conditions, nutrient supply, and light diffusion. Multiparametric studies can easily be carried out through complex distribution networks, valves, light filters, and incorporated electrodes. Owing to these advantages, later microfluidic studies were able to investigate growth kinetics and heterogeneity of single cells as well as optimize the production of pigments or lipids from multiples cell strains with high throughputs. However, in situ analysis of many microalgal metabolites still requires the development of novel miniaturized detection technologies [13]. The feasibility of using microfluidic technologies for optimizing larger scales of microalgae cultivation and commodity production is the focal point of future applications; therefore, this review provides a summary of existing studies and comments toward following research.

In the prospect of microalgae valorization, biorefinery is further required to separate, purify, and/or convert the commodities produced during microalgae culture [14, 15]. Miniaturized downstream processes also have the benefits of mastering the process conditions and performing in situ monitoring of yields and quality of end products. Several attempts have been made to establish microfluidic techniques for biomass concentration, cell weakening, and biomass transformation/fractionation, but more sophisticated techniques are required to gather practical information for commercial-scale applications. In additional to pigments and lipids, microalgae produce a variety of highly valued commodities with potential applications in anticancer/anti-inflammatory treatments, nutritional and pharmaceutical supplements, and upgraded chemicals. However, the production of these microalgal compositions has been rarely investigated in microfluidic platforms. Useful information from related microfluidic studies is summarized and suggestions toward the development of following microfluidic technologies for valorizing microalgae industry are provided in “Future developments”.

## Cultivation of microalgae in microfluidic devices

Unlike the commonly studied biological cells, such as mammalian cells and bacterial cells, microalgae are usually in planktonic state rather than attached state unless suitable environment is provided. Since microalgae are generally non-adhesive cells driven by streams, it is necessary to trap them in the microdevices to be able to study them at cell scale, or follow the same population undergoing a continuous medium flow. The microscale or microfluidic bioreactors can be classified into three categories based on their designs: (1) mechanical traps; (2) droplets; and (3) microchambers (Table 1). Mechanical traps consist of microstructures designed in flow channels to retain cells; droplet systems trap cells in water droplets surrounded by hydrophobic solvents; and microchambers are microreactors where cells are free in an enclosed environment.

**Table 1:**
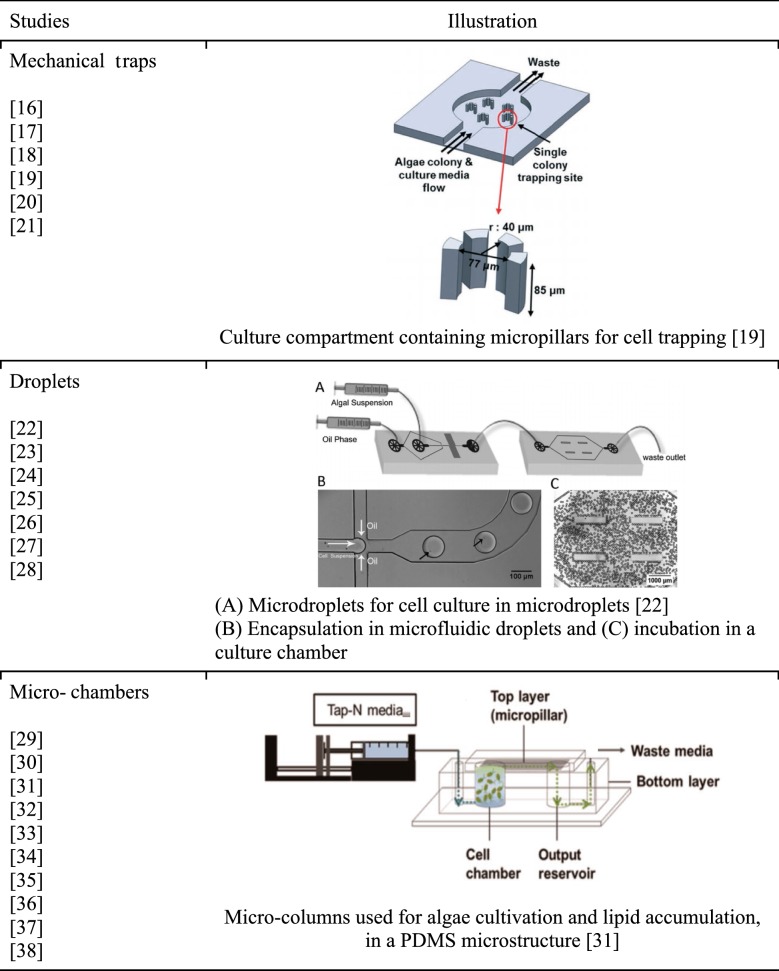
Classification of microfluidic technologies used for cell culture

### Mechanical traps

Generally, mechanical traps enable to study single cells, immobilized in an environment, while a medium could flow. Such traps allow continuous cell monitoring via microscopy. The first demonstration of culturing microalgae in microfluidic devices was performed in 2010 [16], which used physical traps to retain *Botryococcus braunii* in the channels. The trap was composed of four poles arranged into a semicircular pattern with gaps smaller than the size of *B. braunii* cells, resulting in 200 pL available space in the trap for microalgae culture. A C-shaped trap with a culture space of 904 pL is also developed by Bae et al. [17] for the culture of *Chlamydomonas reinhardtii*. These traps randomly capture the microalgae cells infused into the microfluidic device and the cell number retained in the trap is also random. The cross-contamination between traps is also probable when retained microalgae cells overflow after cell division or are flushed out by the hydrodynamic flow. To prevent the unwanted traffic of cells between traps, Eu et al. [18] applied a pneumatic valve at the opening of their 1 nL trap. The peripheral of the trap is surrounded by pillars to enable the perfusion of fresh medium. However, one row of the traps is controlled by the same pneumatic valve; therefore, the exchange of materials between the same row is still possible when one valve is open. Kim et al. [19] reports a microfluidic platform capable of retaining and extracting microalgae cells from a single designated trap. The U-shaped trap has a narrow opening in the bottom to enable flushing cells out of the trap by the hydrodynamic pressure. A valve at the top opening of the U trap is responsible for the selective release of microalgae cells from the trap. Similar to the design in [20], this 15 pL U-shaped trap is also capable of observing and analyzing microalgae cells at single cell level. The growth profile and lipid accumulation of single cell and its subsequent colony for *Botryococcus braunii* and *Chlamydomonas reinhardtii* are included in [16] and [19], respectively. Since the microfluidic trap requires structures or gaps that are smaller than microalgae cells, higher chance of clogging and stricter demand in fabrication precision are anticipated in these devices. Additionally, the extremely low cell density might result in outcomes that deviate significantly from those in bulk experiments as discussed in the later section. A serpentine microchannel connecting traps each with a volume of 27 nL in series is reported by Graham et al. [21]. These traps (600 μm × 600 μm × 75 μm) have sizes much larger than microalgae cells and can retain around 3 × 10^5^ cells to acquire average properties of *Synechococcus elongatus* cultured in the device. Although the design and fabrication are much less demanding than the small traps, the variation of inoculated cells in each trap can be high and it can be challenging to reproduce the tests.

Rather than using physical traps, some reports built microchannels or microchamber with a height slightly inferior to the cell diameter. Luke et al. [22] designed culture chambers of 1.4 mm diameter to grow different microalgae species under continuous medium: *Synechocystis sp*., *Synechococcus elongatus* and *Chlorella sorokiniana*. Different cell chamber heights were adapted to be slightly smaller than the cell width. Chamber height was 1.25 µm for *Synechocystis* cells (1.75 µm average diameter), 0.74 µm for *Synechococcus* cells (1 µm average width), and 3.25 µm for *C. sorokiniana* (estimated 5 µm diameter). Multiple pillars were added in low-height chambers to prevent structure collapse. To prevent phototoxicity from image acquisition, electron-multiplying charged coupled device (EMCCD) was employed. The authors also developed a tracking algorithm able to segment images, identify individual cells, and track growth and fluorescence over time. In a previous study, Min et al. [23] cultivated *Chlamydomonas reinhardtii* cells (about 10 µm diameter) in 2–2.5 µm-height PDMS microchannels. These compressions enable to immobilize the cells during culture and facilitate their monitoring. However, such mechanical stress may impact cell structure and metabolism compared with the physical traps previously described. Also, cells cannot be easily released and recovered from such systems.

### Microfluidic droplets

The use of microfluidic droplets enables to enclose single or multiple cells in an independent environment, and thus can mimic the batch culture conditions. Additionally, droplets allow easy cell sorting and extremely high throughput. Microfluidic droplets have been widely applied to research in multiple areas, such as cell culture (microbial and mammalian), chemical reactions, and protein crystallization [24]. However, the study of microalgae in microfluidic droplets started late in 2011 [25] and the number of publication is small. There are two main techniques for generating microfluidic droplets: continuous flow emulsion and electrowetting [26], and the latter is also called “digital microfluidics”. Generating microfluidic droplets based on continuous flow emulsion is less demanding on the microfabrication and surface treatment compared with electrowetting-based droplets. Droplets can be readily produced by infusing two immiscible fluids (phases) into T junction or flow-focusing microchannels with suitable flow rates [27]. The droplet size and the encapsulated cell number can also be controlled straightforwardly through adjusting the flow rate and the initial cell concentration. Additionally, the throughput of continuous flow droplets can be as high as 1 × 10^6^ min^−1^ [28], while the throughput of electrowetting droplets is limited by the amount of electrodes in the device [29]. The quantitative study in the effects of initial cell number on the proliferation of *Chlamydomonas reinhardtii* is achieved in [25], owing to the access to a sufficient number of droplets containing the same number of microalgae cells. The results indicate that the growth of *Chlamydomonas reinhardtii* depends on both the initial cell number and the droplet size. An initial cell density larger than 1.1 × 10^8^ cells mL^−1^ (or 1 cell in a 268 pL droplet) is required to ensure 60% viability. *Chlamydomonas reinhardtii* are also cultured in microfluidic droplets generated with different channel designs and reagents [30, 31]. Since the droplets are generated continuously, it is challenging to track specific cells during the examination. The in situ observation of microalgae cells in droplets is demonstrated in [32], using hydrodynamic traps to capture droplets. The proliferation profile of single *Chlorella vulgaris* cell and the size distribution of its succeeding cells indicate the highly heterogeneous characteristics of *Chlorella vulgaris* cells cultured in these droplets. The growth rates vary from 0.55 to 1.52 day^−1^ and the difference in cell size can be as high as 10 μm between the largest and smallest cells. It is worthy to note that the sample size in this static droplet platform is limited to the amount of hydrodynamic traps in the device. This problem was overcome by [33], which used micropillar arrays to capture up to 1400 droplets in culture chambers of different heights, including 30 µm, 80 µm, and 100 µm. In addition to image acquisition of droplet generation and cell growth, the authors performed colorimetric analysis of CO_2_ transfer into the microdroplets using hydrogen carbonate indicator.

Although the continuous droplet has advantages of straightforward operation and high throughput, the finite amount of nutrients in the droplet can be consumed rapidly and long-term experiments such as lipid accumulation can be challenging. On the other hand, adding fresh medium or reagents into the electrowetting-based droplets is readily feasible as shown in several reports [34, 35]. The size of electrowetting droplet for culturing *Cyclotella cryptica* ranges from 10 to 70 μL in these studies. Small droplets are divided from reservoirs containing medium or fluorescence dyes and transported to the droplet containing microalgae by changing the dielectric properties of the dielectric layer on the electrode through applying an external voltage [36]. However, precautions should be taken when applying multiple reagents, since the residue of reagents on the path can raise the concern of cross-contamination [37, 38].

Finally, Wang et al. [39] developed an original method using the surface of an air bubble formed in an aqueous solution to isolate microalgae cells. The air bubble is controlled with a digital syringe to create a water/gas interphase at a T junction. The effects of pH variations on the captured cell, *Dunaliella salina* and *Tetraselmis Chui*, were then studied by injecting NaClO or formaldehyde into the channel. Cell capturing was, however, affected by pH, because an increase in pH triggers positively charged ions precipitation which neutralizes the negative charges surrounding microalgae cells.

### Microchamber

Microchambers can be considered as downscaled photobioreactors, in which a cell population is cultivated. Culture scale is generally larger than the previous microfluidic devices and enables to perform analysis based on biomass and to get closer results to bulk culture conditions. The first microchamber designed for microalgae study was presented in [40], in which a microfluidic device made of hybrid PDMS/glass to culture *Tetraselmis chuii* and *Neochloris oleoabundans* was built. The culture chamber, 17.5 mm in length and 2.5 mm in width at the center (total volume of 2.4 μL), was surrounded by a PMMA construct containing torque-actuating screws to seal the chamber. This system enabled to concentrate microalgae cells in the chamber by partially closing the exit valve, or to close the system from exchange for 3–27 days. Microfluidic devices were kept in a sealed polycarbonate container with a transparent lid with pure water vial to avoid evaporation. Lipid accumulation in *Neochloris oleoabundans* was monitored with BODIPY staining. The strong adhesion of these cells on the glass surface enabled to easily shift the surface containing microalgae cells to perform fluorescence imaging and observe lipid accumulation in nitrogen-depleted *Neochloris oleoabundans* cells.

The volume of later microchamber devices ranges from 40 to 400 μL and they are usually designed to fit the format of commercial plate reader for straightforward observation. Several designs are available including stand-alone microcolumns with one inlet and one outlet [41–43], microcolumns connected in series [44], and microcolumns with multiple inlets for multi-stress tests [45]. Strictly speaking, the dimensions of these microcolumns exceed the scale of microfluidics. However, they are connected with microchannels or microfilters (composed of micropillars) and are easily adaptable to investigations of various processes in microalgae biotechnology. Therefore, they are worthy of great attention. The 40 μL microcolumns connected in series by a straight microchannel were developed by Perin et al. [44]. The continuous infusion of fresh medium through the microchannel guarantees that the growth and metabolism of *Nannochloropsis gaditana* are not limited by the amount of nutrients. They also found that the amount of CO_2_ in the microcolumn was sufficient for *N. gaditana* in the microcolumn owing to the high permeability of CO_2_ in the thin PDMS cover. However, the size of the microchannel (500 μm) is much larger than microalgae cells and the dilution of culture is inevitable. This can lengthen the duration before stationary phase and is undesirable when investigating the induction of lipid accumulation. Therefore, outlet microchannels having size (2 μm) smaller than microalgae cells [42, 43] and microfilter composed of micropillars [41, 45, 46] are applied to retain microalgae cells in the column. These features also enable in situ extraction of lipids for further analysis as addressed in a later section. It should be noted that since the volume of microcolumns is comparable to that in the multiwell plate, the sedimentation of microalgae cells is likely and agitation or mixing is required for homogeneous culture condition and accurate optical measurements.

### Comparison of microalgae culture in different microfluidic systems

Growth rates of microalgae cells are the most intensively studied topics in microfluidic bioreactors, because they can be straightforwardly estimated by cell counting or optical density measurements. Comparing the growth rates of microalgae strains in different microsystems (Table 2), the size of which varies from hundreds of picoliters to hundreds of microliters, can be an efficient way to evaluate these culture devices regarding nutrients, light, and CO_2_ supply. For *Chlamydomonas reinhardtii* cultured in different microfluidic devices, consistent results from five studies [18, 19, 23, 25, 30] were obtained with doubling time ranging from 6 to 10 h, corresponding to growth rate of, respectively, 2.77 day^−1^ to 1.66 day^−1^. The highest growth rate was obtained using single cell trapping system, supplied with continuous TAP medium, and lighting conditions of 100 µmol m^2^ s^−1,^ supplied as 12 h/12 h light dark cycle [19]. A deeper trap [17] displays slightly lower growth rates, which can be explained by the higher number of cells in each trap, where slight shading effects might happen. Single cells cultivated in microdroplets [30, 45] enabled to obtain growth rates comparable to single cell trapping. The microchambers [23, 45] produced slower growth rates, ranging from 0.7 to 1.7 day^−1^, in comparison with single cell mechanical traps or droplets. Similarly, for *Chlorella vulgaris*, the growth rates measured were higher in microdroplets, from 1.39 to 2.3 day^−1^ [32, 33, 47], than in microchambers, from 0.71 to 1.2 day^−1^ [45, 48]. The difference in growth rates was also observed for *Chlorella sorokiniana* cultivated in static droplet, 2.8 day^−1^ [33], and chamber, 1.75 day^−1^ [22]. For *Neochloris oleoabundans*, the growth rate was measured at 2.85 day^−1^ in the droplet [33], and 1.1 day^−1^ in the chamber [45]. For *Synechococcus elongatus,* the growth rate was measured much higher, 2.28–2.92 day^−1^, in a thin (0.74 µm height) static chamber [22], compared to a thick (75 µm height) chamber (0.8 day^−1^) [21]. Although one can observe an inverse correlation between the size of the microsystem and the measured growth rate, many parameters should also be considered: the light transmission through the microsystem (PDMS might attenuate light), CO_2_ supplement and its diffusion rate into the device, medium, and temperature. For example, most droplet systems applied acetate in the TAP medium as the carbon source for *Chlamydomonas reinhardtii,* while microchamber systems applied carbon dioxide. The heterotrophic culture of *Chlamydomonas reinhardtii* is reported to have higher growth rate than the autotrophic culture [49, 50] and this can also contribute to the higher growth rates of *Chlamydomonas reinhardtii* in the droplets.

**Table 2:** Comparison of growth rate obtained for different strains in microsystems

Strain	Technology	Culture size	Growth rate (day^−1^)	Conditions	Refs..
*Chlamydomonas reinhardtii*	U-shape trapping	75 µm × 16 µm × 15 µm	2.08–2.77	100 µmol m^2^ s^−1^ 12 h dark cycle TAP medium	[20]
	Trapping chambers	60 µm radius, 30 µm height	0.45–1.34	120 µmol m^2^ s^−1^ TAP medium	[17]
	Perfusion chamber	200 µmx100 µmx30 µm	1.85–2.08	Microscope halogen lamp TAP medium	[18]
	Compressing channel	100 µm width, 2–2.5 µm height, 20 mm length	1.39	TAP medium	[32]
	Microdroplet	40 µm radius 268 pl/droplet	2.07	55 µmmol m^2^ s^−1^ TAP medium	[22]
	Static droplet	120 µm radius	1.39–2.7	80 µmol m^2^ s^−1^ 12 dark cycle TAP medium	[28]
	Flowed droplets	330 µm radius 150 nl/droplet	1.51–2.37	20 µmol m^2^ s^−1^ 460 and 650 nm LED TAP medium	[26]
	Microchamber	500 µL	0.7–1.1	23 °C with 5% CO_2_ Tris–phosphate medium	[35]
*Chlorella vulgaris*	Microdroplet	40 µm radius 268 pl/droplet	1.39	55 µmmol m^2^ s^−1^ BBM medium	[22]
	Static droplet	45 µm radius (uncompressed)	1.8–2.3	Ambient—7.5% CO_2_ 35–200 µmol m^2^ s TAP medium	[27]
	Static droplet	134 µm radius 10 nl/droplet	1.52	905 lx 8 h dark cycle +glucose	[24]
	Microchamber	500 µL	1.0 - 1.2	23 °C with 5% CO_2_ Tris–phosphate medium	[35]
*Chlorella* sp.	Microchamber	1.2 mm × 2 mm × 100 µm	0.71	80 µmol m^2^ s^−1^ F/2 medium	[30]
*Chlorella protothecoides*	Static droplet	45 µm radius	3.14	Ambient—7.5% CO_2_ 35–200 µmol m^2^ s TAP medium	[27]
*Chlorella sorokiniana*	Static droplet	45 µm radius	2.80	Ambient—7.5% CO_2_ 35–200 µmol m^2^ s TAP medium	[27]
*Chlorella sorokiniana*	Static chamber	3.25 µm height 1.4 mm ring shape	1.75	100 µmol m^2^ s 5% CO_2_ BG11 medium	[38]
*Chlorella cryptica*	Microdroplet	70 µL droplets	0.39	f/2 medium 14 °C 60 W lamp	[23]
*Dunaliella tertiolecta*	Microdroplet	40 µm radius 268 pl/droplet	0.69	55 mmol m^2^ s^−1^ Specific medium	[22]
*Neochloris oleoabundans*	Static droplet	45 µm radius	2.85	Ambient—7.5% CO_2_ 35–200 µmol m^2^ s TAP medium	[27]
*Neochloris oleoabundans*	Microchamber	4 mm × 2 mm × 3 µm	1.0–1.2	23 °C with 5% CO_2_ Tris–phosphate medium	[35]
*Nannochloropsis gaditana*	Microwells	40 µL wells 2.1 mm radius	0.25–0.5	60 mmol m^2^ s^−1^ F/2 medium	[37]
*Platymonas subcordiformis*	Microchamber	1.2 mm × 2 mm × 100 µm	0.31	80 µmol m^2^ s^−1^ F/2 medium	[30]
*Porphyridium cruentum*	Microchamber	1.2 mm × 2 mm × 100 µm	0.52	80 µmol m^2^ s^−1^ F/2 medium	[30]
*Platymonas helgolandica*	Microchamber	1.2 mm × 2 mm × 100 µm	0.75	80 µmol m^2^ s^−1^ F/2 medium	[30]
*Phaeodactylum tricornutum*	Microchamber	1.2 mm × 2 mm × 100 µm	1.52	80 µmol m^2^ s^−1^ F/2 medium	[30]
*Synechococcus elongatus*	Static chamber	0.74 µm height 1.4 mm ring shape	2.28–2.92	50–100 µmol m^2^ s^−1^ 5% CO_2_ BG11 medium	[38]
	Microchamber	600 µm × 600 µm × 75 µm	0.8	5 - 148 µmol m^2^ s^−1^ BG-11 medium	[21]
*Synechocystis*	Static chamber	1.25 µm height 1.4 mm ring shape	0.73	20 µmol m^2^ s 5% CO_2_ BG11 medium	[38]
*Scenedesmus* sp.	Microchamber	4 mm × 2 mm × 3 µm	0.4	5% CO_2_ Tris–phosphate medium	[35]

Single cell tracking using mechanical traps may be the best choice to monitor individual microalgae cells in optimal growth conditions, since there would be negligible limitations for nutrient or light in comparison with other devices. The possibility of continuously supplying fresh medium enables to easily switch from growth to stress. Single cell trapping also enables to accurately monitor various cells displaying different morphologies and cytoplasmic contents among the same microalgae population. In addition to separating the cells individually, traps also enable the cell immobilization during the whole measurement process for the precise single cell analysis. This technique may be the best choice to study and understand microalgae at single cell scale; however, it may become unfavorable regarding fabrication costs of traps per cell and the difficulty to scale up to large-scale production.

As previously discussed, EWOD manipulation can be the most efficient method for reagent handling. This technology may be specially adapted for applications concerning liquid/liquid transfers such as cell labeling and metabolites extraction. However, upscaling such systems for cell culture seems to be economically inappropriate. Microfluidic flow droplets (emulsion) may be especially convenient to generate multiple closed environments and mimic batch cultures using one or plural cells encapsulated in a culture medium. This technology may be the best choice to study the effects of chemical environments on the morphology, growth kinetics, and/or monitor the dividing behavior of a mother cell into daughter cells. The straightforward integration of flowing droplets and flow cytometry/fluorescence-activated sorting makes it a powerful tool for screening and selecting the desired cells/strains. However, scaling up also seems inappropriate because of the difficulty in recovering the biomass from the droplets without high energy expense.

In contrast to the above microfluidic cultures, microchambers may be inappropriate for monitoring single cells, but these structures may, however, be particularly useful to test different designs, conditions, and evaluate the productivity before scaling up the process. These systems may also be scaled up and stacked to large production, finding the right compromise between productivity and costs. It should be noted that small scaled chambers would lead to high productivity with reduced nutrients and light limitations, but increased costs due to fabrication and hydrodynamic pressures.

## In situ measurement

A multiplicity of detection techniques might be incorporated inside microdevices to monitor the cell growth, viability, or lipid contents. Usual characterization methods employed for pilot-scaled cultures are generally unsuitable for these microscaled reactors (from nanoliter to hundreds of microliters working volumes). Novel techniques must be developed to fit these restricted volumes. The main techniques developed for microscale microalgae culture can be separated into optical and electrical characterizations.

Optical analysis requires illuminating cells with a light source (LED or laser) and to recover the signal with a photosensor. Mirrors and filters could be necessary to guide and treat the light. Fluorescent dyes can be used to stain specific microalgae features (DNA, lipids, membrane, cell wall, enzymatic activity, etc.) [51]. Bright field imaging enables the direct observation of cells, but post-process imaging must be carried out to classify cell characteristics. Hu and Davis [52] developed the automatic image processing of diatoms with dual classification according to their shape and texture. Instead of recording raw images, light scattered from laser-excited cells measured with PMT detectors was applied to classify cells according to their size, shape, or internal properties such as organelle densities [53]. Schaap et al. [54] also measured light diffraction to differentiate five microalgae species using a quadrant-cell detector which monitored very small intensity changes after exiting the microchannel. A red laser wave guide was integrated at the exit of the channel and the acquired data were correlated to particle imaging recorded at 120 fps.

**Fig. 1:**
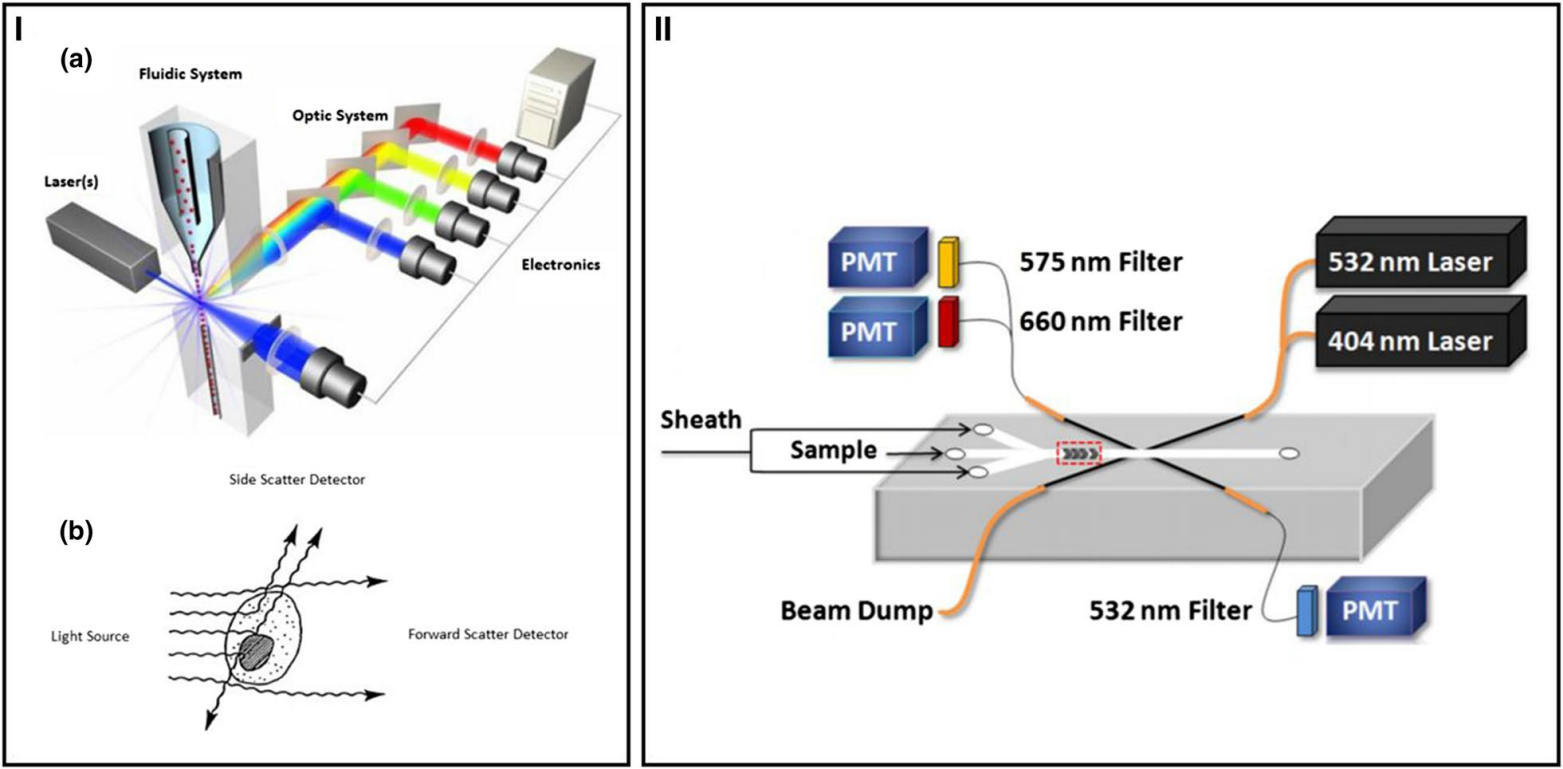
Use of flow cytometry. **I** Illustration of the principle. (a) Basic components of a flow cytometer; (b) light scattering properties of a cell [147]. **II** Integrated flow cytometry in a microsystem for algae fluorescence detection [56]

Light diffraction is highly useful in acquiring information related to microalgae morphology; however, fluorescence measurements are preferably performed for microalgal photosynthetic activities and lipidomic metabolites. Most of the microfluidic systems use blue laser (470–490 nm) as illumination and a sensor collecting red light (630–675 nm) to measure chlorophyll contents. The optical setup for continuous flow microfluidic analysis consists of classical components and follows principles for a common flow cytometry as shown in Fig. 1. Benazzi et al. [55] integrated 532 nm and 633 nm lasers into a channel through a beam expander and objective lens to illuminate microalgae, and fluorescence was collected through detectors with different filters. The authors were able to identify three types of microalgae species in a sample of 2500 cells with comparable results to a commercial cytometer. Hashemi et al. [56] used 404 nm and 532 nm guided lights through an optical fiber into a microfluidic channel to analyze three microalgae. Chlorophyll and phycoerythrin fluorescence were, respectively, measured at 660 nm and 575 nm. Results showed that elongated cells, such as *Pseudo*-*Nitzschia*, may enter in the microchannel at different angles and produce various light-scattering angles that affect the signal homogeneity. A slow flow rate of 10 µL min^−1^ enables an efficient identification in comparison with 200 µL min^−1^. To decrease the size of the optical setups, light-emitting diodes (LEDs) and photodiodes/photomultiplier tubes (PMT) were applied to replace lasers and CCD/CMOS sensors attached to the microscope. Damodaran et al. [30] used a blue LED (470 nm) focused with a 20× objective lens to illuminate a fluorinated ethylene propylene (FEP) tube containing microalgae droplets, and the emitting light was collected using a set of dichroic mirrors, an emission filter (660 nm), and a PMT tube. Fluorescence intensity measured in each droplet was correlated to a cell number of *Chlamydomonas reinhardtii* and the method was compared with external flow cytometry with similar results. Wang et al. [57] integrated a photosynthetic sensor into a fluidic channel with a 488 nm laser diode (used power 2-8 mW) to illuminate the cells and a photodiode to detect the chlorophyll autofluorescence. They were able to distinguish the living cells of five microalgae species. The same group [58] used an excitation laser at 488 nm and a photomultiplier equipped with a filter 680/40 nm to detect the chlorophyll activity of immobilized cells. Chlorophyll activity kinetics was estimated from relative fluorescence intensity before and after cell treatment. Best et al. [59] used fluorescence measurements to sort droplets containing cells (positive droplets) by applying a voltage pulse (700 V) at the entrance of the channel junction. Lasers and photomultipliers were adapted to *Chlamydomonas reinhardtii* (ex: 480 nm, em: 635 nm LP) and cyanobacteria (ex: 594 nm, em: 629/30 nm). Nitrogen-depleted *Chlamydomonas reinhardtii*, i.e, with low fluorescence intensity, were sorted at 160 Hz and resulted in 91% positive droplets containing cells. Lefèvre et al. [60] incorporated an organic photodetector (OPD) made of two 50 nm stacks of blue (480 nm) and green (515 nm) organic light-emitting diodes (OLEDS) in a microfluidic chamber to monitor *Chlamydomonas reinhardtii* fluorescence, and found a correlation between fluorescence and cell concentration. OLEDS and OPD may provide new advances in microalgal detection, thanks to miniaturized systems and easily tunable fluorescence sensors [61]. In addition to autofluorescence measurements (pigment detection), cell viability can be evaluated from enzymatic activities with the use of fluorescein diacetate (FDA). Zheng et al. [48] injected 20 µg mL^−1^ FDA into microchambers to detect *P. cruentum* enzymatic activity and obtained similar viabilities with a large-scale culture.

In the perspective of biofuel production, intracellular lipid droplets can be stained using lipophilic dyes for flow-through fluorescence detections [62]. Nile red was the most commonly used dye in earlier studies, but BODIPY has become more favorable for microscopic or flow cytometric measurements since it has a higher specificity toward neutral lipids, which can be transesterified to biodiesel. However, Holcomb et al. [40] reported that on-chip staining with BODIPY dye was not ideal due to its strong absorption onto the PDMS portion of the microchip. In fact, all hydrophobic dyes share the same concern of high adsorption rate, which leads to extremely high fluorescence background when performing on-chip labeling. Therefore, additional efforts are required to achieve high-quality fluorescence detections when on-chip labeling is involved. Removing excessive hydrophobic dyes using liquid–liquid extraction has been accomplished in [63], and the signal to noise ratio of the fluorescence detection for Nile red labeled lipids inside *Chlorella vulgaris* was increased by 17-fold. Kim et al. [31] applied similar principles to remove excessive Nile red from the sample with a more sophisticated microfluidic device capable of generating droplets containing *Chlamydomonas reinhardtii* cells or Nile red, merging the aforementioned droplets and washing the merged droplets with fresh oil. Rinsed droplets were then collected in an observation chamber to quantify oil production with fluorescence microscopy. Nile red fluorescence was measured with a yellow channel (ex 460–500 nm/em 560–600 nm) and chlorophyll fluorescence with a red channel (ex 460–500 nm/em 610 LP). Except for using liquid–liquid extraction to remove excessive dyes, Shih et al. [34] used electrowetting on dielectric (EWOD) droplet manipulation to deliver a lipid-sensitive dye (LipidTOX) to microalgae droplets with respect to a precise ratio dye quantity per cell. Automatic manipulation enabled illuminating single droplets containing microalgae culture, carry them to absorbance (chlorophyll) and fluorescence (stained lipids) measurements, and repeat measurement cycle several times on the same droplets, realizing up to 30-fold reduction in manual intervention.

**Fig. 2:**
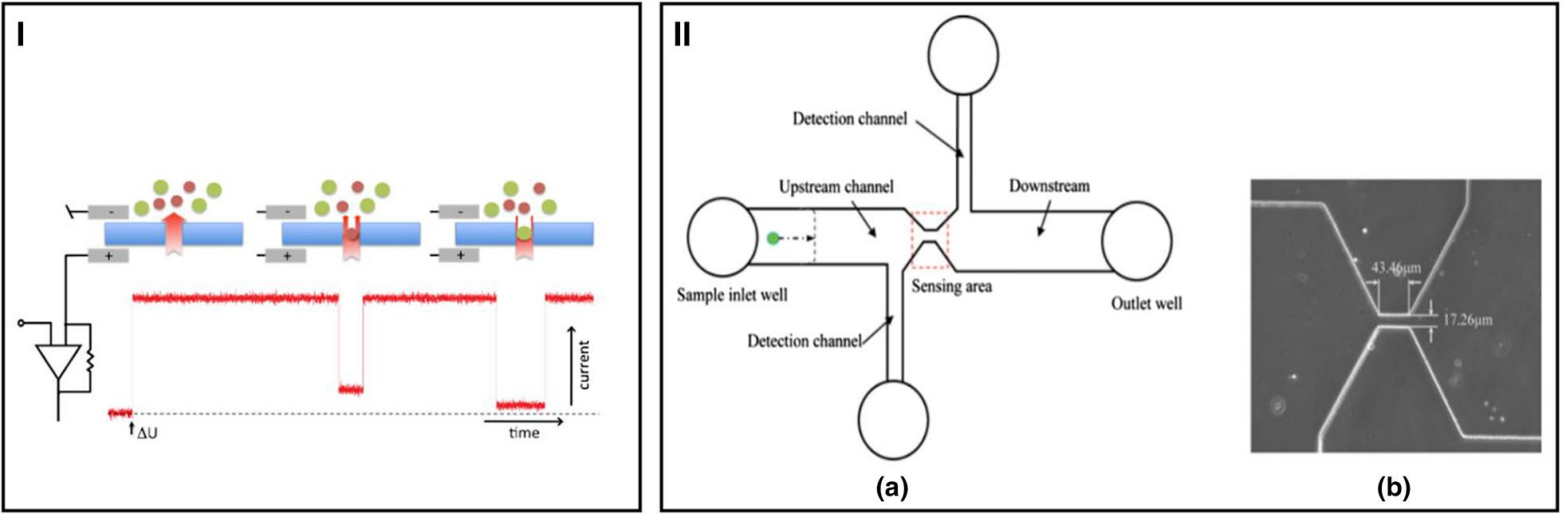
Resistive gate sensor for algae counting and sizing detection. **I** Principle [148]. **II** Algal detection system [64]

In addition to optical measurements, electrical characterizations can also be used to detect microalgae properties. Song et al. [64] used a resistive pulse sensor (RPS) to monitor cell number and size by integrating small gates (43.46 µm wide, 17.26 µm long, 25 µm high for *Pseudokirchneriella subcapitata*; 5.93 wide, 34.57 long, 5 µm high for *Chlorella vulgaris*) in PDMS channels (Fig. 2). Similar RPS was applied in [65] to estimate cell size and to distinguish live cells to lysed cells. The same group also developed capacitive detection of microalgae in a microchannel in the range of 200–500 kHz and observed a shift of capacitive response between live and dead cells [66]. Benazzi et al. [55] estimated cell size (discriminating cell sizes from 2, 3, and 4 µm) using impedance spectroscopy in a microchannel (300 kHz–6 MHz). Although the design and fabrication of microelectrodes are straightforward for these systems, the accuracy of the measurement strongly depends on the fraction of cells between the electrodes and the compositions of the medium applied for the measurement. Therefore, closely arranged electrodes and sample pretreatment to control the medium composition are necessary.

**Fig. 3:**
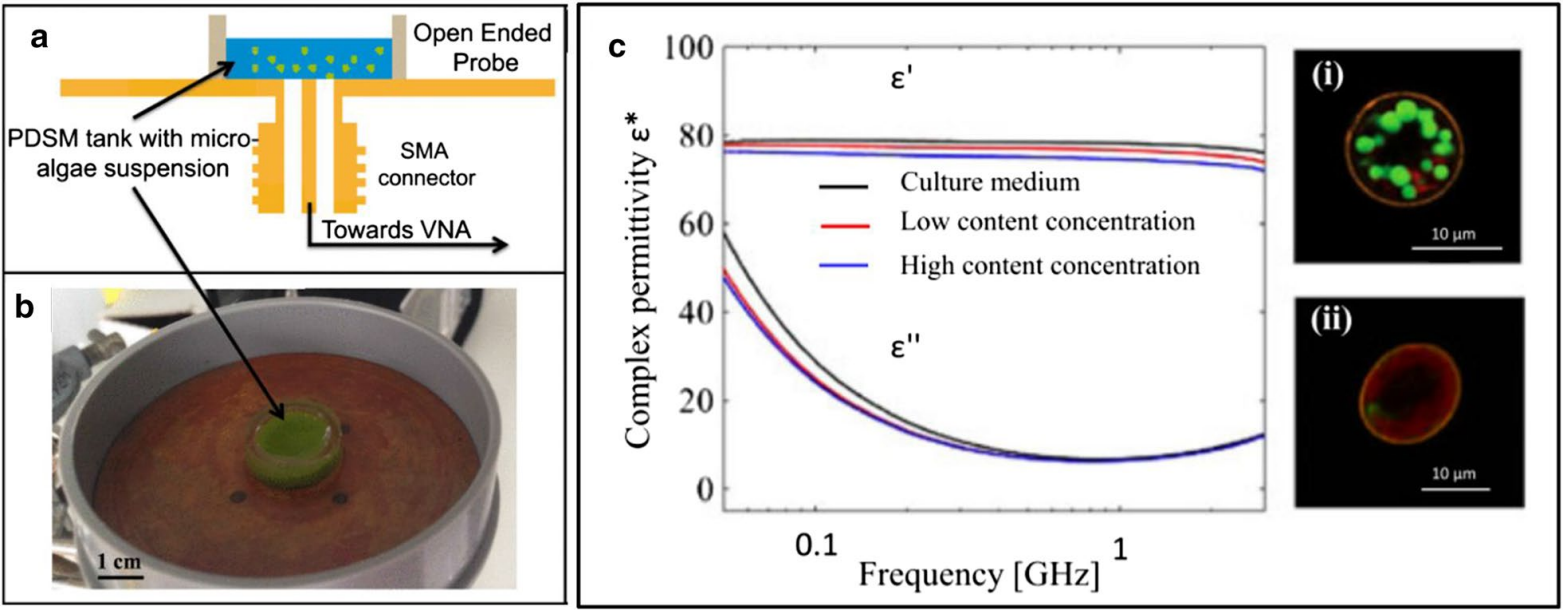
Lipid biosensor. **a** The sensor is based on a coaxial line and a modified connector sealed at the reservoir side (**b**) for microalgae suspension characterization. **c** Frequency-dependent complex dielectric permittivity for different cellular lipids content. Images of confocal laser scanning microscopy showing *Chlamydomonas reinhardtii* cells: (i) high lipid content (15%), (ii) low lipid content (3%) [68]

On the other hand, analyses based on dielectric properties are not affected significantly by the fraction of cells in the sample; moreover, dielectric characterization of microalgal cells may enable characterizing the intracellular lipid abundance. Bono et al. [67] observed different dielectric behaviors of *Chlamydomonas reinhardtii* cells with various lipid abundances due to a decrease in the cytoplasm conductivity. Fellahi et al. [68] developed a lipid quantification biosensor using dielectric spectroscopy at radio frequencies (30 MHz–3 GHz) based on the slight decrease of dielectric permittivity of microalgae suspension when the lipid content increases (Fig. 3). Dielectric properties can also be used to sort cells with different cellular compositions. Hadady et al. [69] separated cells depending on their lipid abundance at 41 MHz and the same group also observed a shift in the DEP crossover frequency, from 75 to 40 MHz, in lipid-accumulating cells [70]. Deng et al. [71] were able to separate microalgae depending on their lipid abundance at a frequency of 20 MHz and a medium conductivity of 2.95 ms/cm. Gallo-Villanueva et al. [72] developed insulator-based dielectrophoresis (iDEP) by applying direct current electric field (ranging from 500 to 1200 V/cm) in a channel containing 32 cylindrical insulating posts. Experiments showed that live and dead cells had different electrical attractions to the post and could be spatially separated.

## Screening of cultivation conditions

The advantages of high throughputs and small sample/reagent amounts of microfluidic platforms make them favorable tools for exploring the optimal conditions for microalgae cultivation. The cultivation parameters that have been investigated in microfluidic platforms include lighting conditions (light intensity, duty cycle, spectral composition), pH, temperature, salt concentrations (NaCl), CO_2_ concentrations, and nutrient concentrations (acetate, nitrate).

### Lighting conditions

**Fig. 4:**
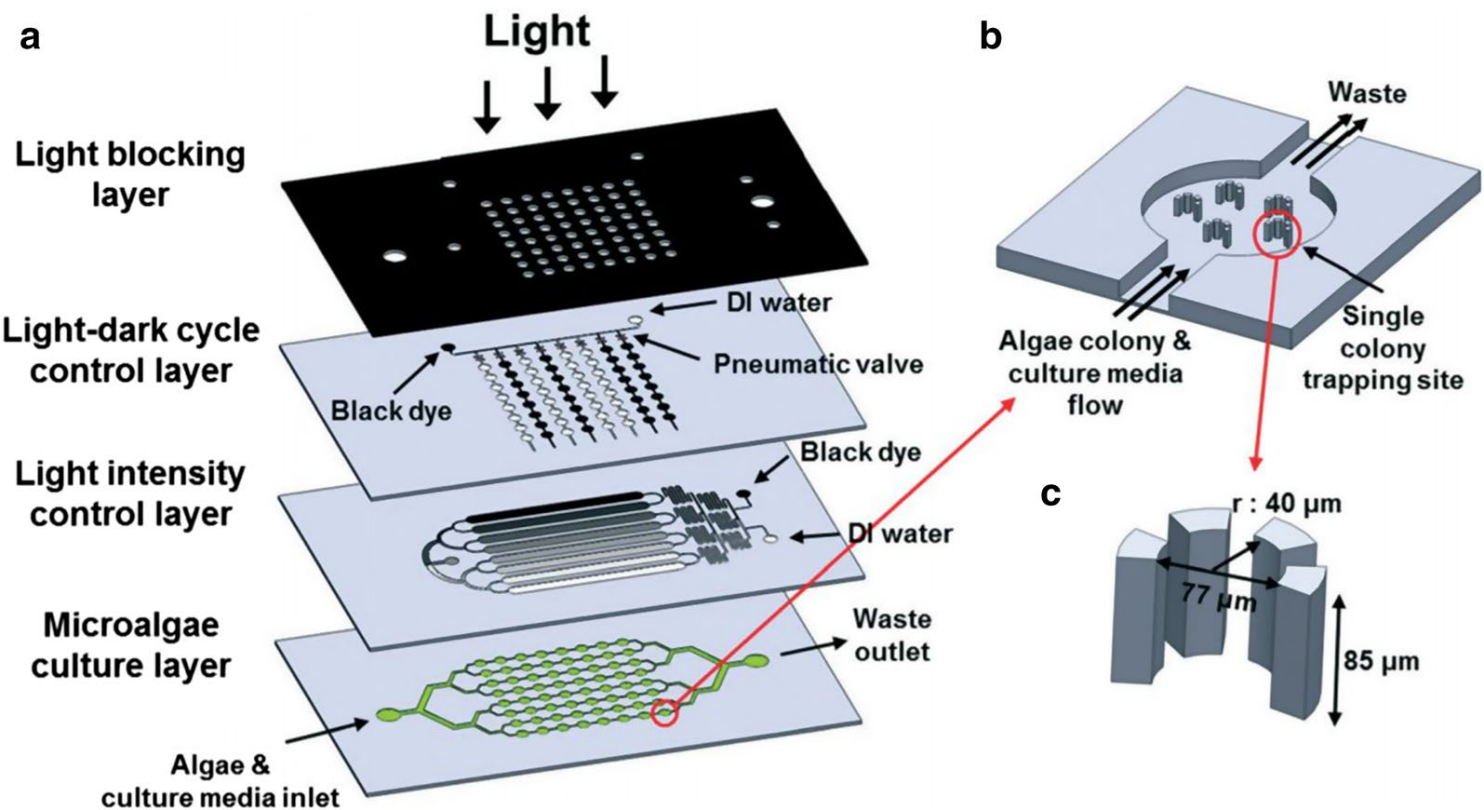
The high-throughput microfluidic microalgal photobioreactor array. **a** The platform was composed of four layers—a light blocking layer, a microfluidic light–dark cycle control layer, a microfluidic light intensity control layer, and a microalgae culture layer [20]. **b** Enlarged view of a single culture compartment having five single-colony trapping sites. **c** A single-colony trapping site composed of four micropillars

**Fig. 5:**
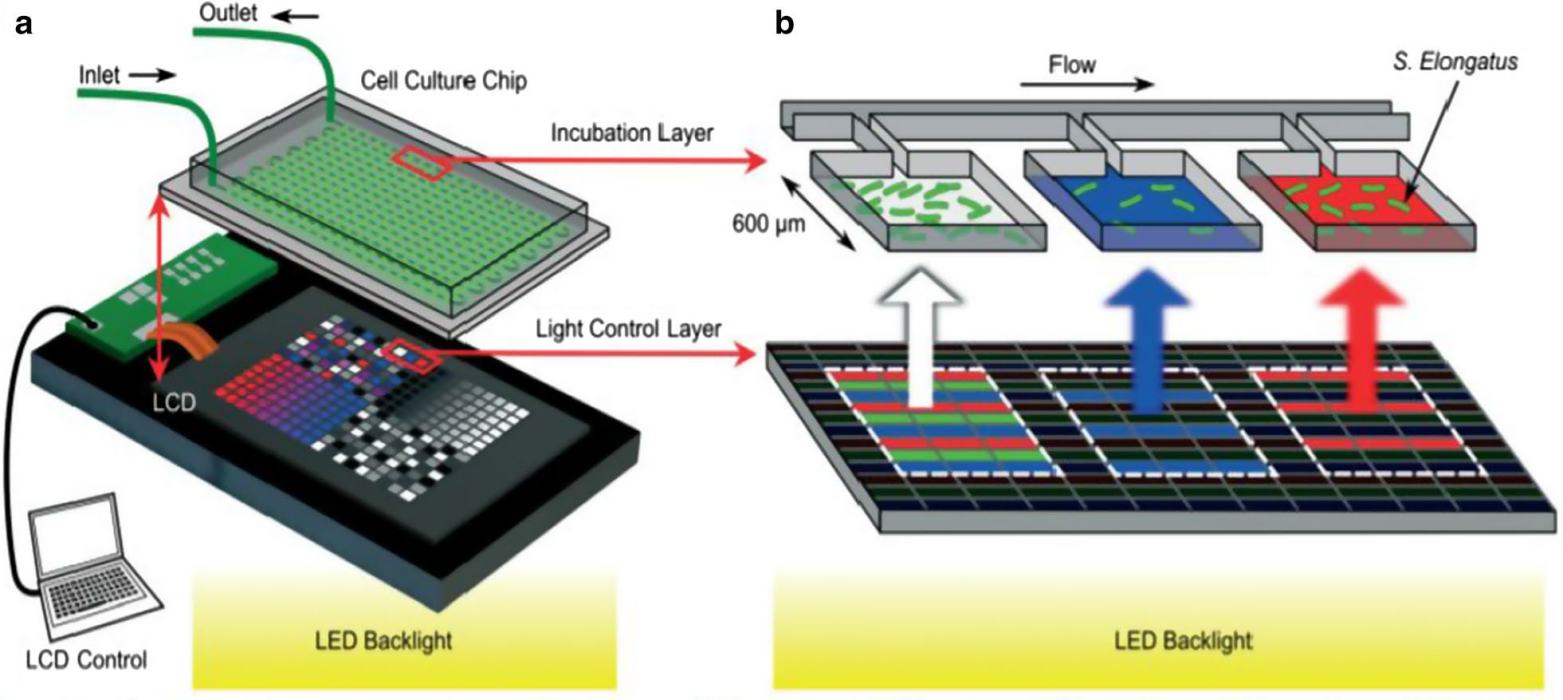
Screening of intensity, time variance, and spectral composition of irradiance on 238 microreactors [21]. **a** Schematic of the multiplexed pixel-based irradiance platform, consisting of a PDMS-on-glass cell culture chip, a programmable LCD screen and an LED array backlight. **b** Pixels directly below each incubation microreactor are individually controlled to project the desired irradiance. The irradiance intensity, time variance and spectral composition are each tuned based on experimental requirements. The PDMS is illustrated as transparent for clarity; in all experiments it is cast black (opaque) by adding graphite

Since the volume of microfluidic microalgae cultivation is small, the self-shading effects of light are minimum and enable the accurate analysis of cellular response toward lighting conditions. Therefore, the applications of microfluidic platforms for optimizing lighting conditions have rapidly increased in the past 3 years. A previous review article [4] provides detailed information for the design and fabrication of the microfluidic photobioreactor. The simplest method of applying different lighting conditions to microfluidic devices is placing the whole device into a light-controlled environment [42, 43]. Since most of the microfluidic devices for microalgae culture are made of transparent materials (PDMS and glass slides), the light intensity inside the microfluidic compartment should be nearly identical to the imposed intensity. Moreover, PDMS is unlikely to cause light dispersion, since it has nearly identical refraction indexes for different wavelengths of visible lights [73]. To create different light intensities on the same device, actual filters [44] or extra layers of microfluidic channels containing fluids with different dye concentrations [20] can be applied on top of the culture area (Fig. 4). For more sophisticated manipulation of lighting conditions, an LED array [34] or a programmable LED screen with an array of LED backlight [21] can be applied (Fig. 5). The LED array contains diodes with fixed emission wavelengths and easily adjustable duty cycles. It is applied to investigate the growth and lipid production of *Cyclotella cryptica* in the electrowetting-based microdroplet. Results show that the blue light (~ 450 nm) promotes the growth of *C. cryptica*, while the yellow light (~ 580 nm) enhances the accumulation of lipids. The microfluidic experiment also enables them to observe the relationship between light wavelengths and the generation of reactive oxygen species (ROS) for investigating the wavelength-dependent lipid accumulation. Their results suggest that the accumulation of lipids is highly related to the increased amount of ROS. It is possible that *C. cryptica* cannot produce antioxidants under yellow light and cope with the oxidative stress by accumulating lipids. The programmable LED screen with the LED array backlight provides more flexible adjustments toward wavelengths and intensities. Over 30 combinations of spectral compositions and light intensities are examined in [21], and the results indicate that *Synechococcus elongatus* has the highest growth rate at a light intensity of 42 μmol m^−2^ s^−1^ and a spectral composition of ~ 90% red hue (ratio between red and the full spectrum).

The optimal light intensity leading to the highest growth rates in microsystems was found to vary significantly (42–360 µmol.m^−2^ s^−1^) depending on the studies. The differences are likely attributed to the various microalgae strains and the spectral compositions. Additionally, the optimal lighting conditions change when different metabolites are desired. For example, the production of lipids in *Neochloris oleoabundans* [42] and astaxanthin in *Haematococcus pluvialis* [43] requires significantly different lighting intensities in the same microfluidic bioreactor.

### Environmental factors

Except for the lighting conditions, several parameters including pH, temperature, nutrients, and salt concentrations have also been investigated for increasing final cell amounts and pigment/lipid contents in microfluidic studies. The first attempt is accomplished by [47], in which microfluidic droplets (continuous flow, emulsion based) containing different initial pH values, NaCl concentrations, and NO_3_^−^ concentrations are generated and stored for as long as 11 days for cell number quantification under a microscope. This study demonstrates the feasibility of optimizing cultivation parameters in microdroplets by validating similar optimal pH values and NaCl concentrations for *Dunaliella tertiolecta* cultured in the microfluidic droplets and larger-scale cultures. They also investigated the effects of initial NO_3_^−^ concentrations in the droplet on the growth rate of *Chlorella vulgaris* and found that insufficient NO_3_^−^ concentration can decrease the final cell number to as low as 50% of that in the nitrate sufficient droplets. The high throughput of continuous flow microdroplet (60 droplets per second) makes it a great tool for investigating short-term tasks such as growth rate with statistical analysis. However, the evaporation of water in the droplet as well as the consumption of nutrients by the microalgae cells can change the pH value and NO_3_^−^ concentration dramatically during long-term cultivation.

Screening cultivation conditions in closed systems presents the problem of condition drifts such as pH, temperature, nutrient depletion or toxic metabolic by-products. Therefore, optimization of culture conditions for microalgae in microfluidic devices with continuous supply of fresh medium seems a better choice for obtaining results that are more applicable in scaling up or long-term cultivation. The studies conducted by [41–43] provide a simple, yet effective method for investigating the effects of combinations of nutrient composition (pH, NO_3_^−^, NH_4_^+^) and environment conditions (lighting, temperature, CO_2_). Each of the microcolumns received the fresh medium with fixed nutrient compositions from an external source (syringe pump) and as many as 16 microcolumns were placed on the same device. The device was then placed in an incubator with a specific combination of lighting, temperature, and CO_2_ concentration. Each screening took up to 7–14 days depending on the microalgae strain (*Neochloris oleoabundans, Haematococcus pluvialis*) and the targeted metabolites (lipids, astaxanthin). The optimal conditions for lipid production are 5% CO_2_ (v/v), pH 7.5, and 7 mM NO_3_^−^ while those for astaxanthin production are 7% CO_2_ (v/v), and pH 7.0. Although the throughput of these microcolumn bioreactors is comparable to those using Erlenmeyer flasks or well plates, its ability in continuous supply of nutrients creates an environment mimicking that in larger-scale continuous process. Moreover, the sampling of effluent from the microcolumn is straightforward, because microalgae cells are retained in the microcolumn by the filter or narrow microchannels. Since the infusion rate is around 100 μL min^−1^, sufficient amounts of effluent can be collected and analyzed by conventional methods such as HPLC and UV spectrometer for acquiring the change in nutrient compositions during the cultivation. However, the number of syringe pumps required for each screening can be as high as the number of microcolumns in these devices and this makes the operation even more economically costly than conventional screening using flasks and well plates. To solve this issue, the same group developed a microfluidic device containing eight microcolumns that share the same inlet for the fresh medium for screening the effects of multiple stresses (nutrient starvation, high salt, high temperature, pH shift) on the lipid production in six strains of microalgae [45]. By combining more than one stress in the medium, the synergistic effects of different stresses on the lipid production can be identified. They conclude that combinations of two stresses generally result in higher lipid productivity than single or more than two stresses. The highest lipid productivity of 167 mg L^−1^ day^−1^ is achieved by imposing 200 mM NaCl and N-starvation on *Chlorella protothecoides*. Multiplexed results are obtained from each device which requires only one syringe pump. The adjustment of medium compositions such as switching from nitrogen-sufficient medium to nitrogen-depleted medium can be accomplished as simple as switching the medium in the syringe or applying microfluidic dilution techniques.

**Fig. 6:**
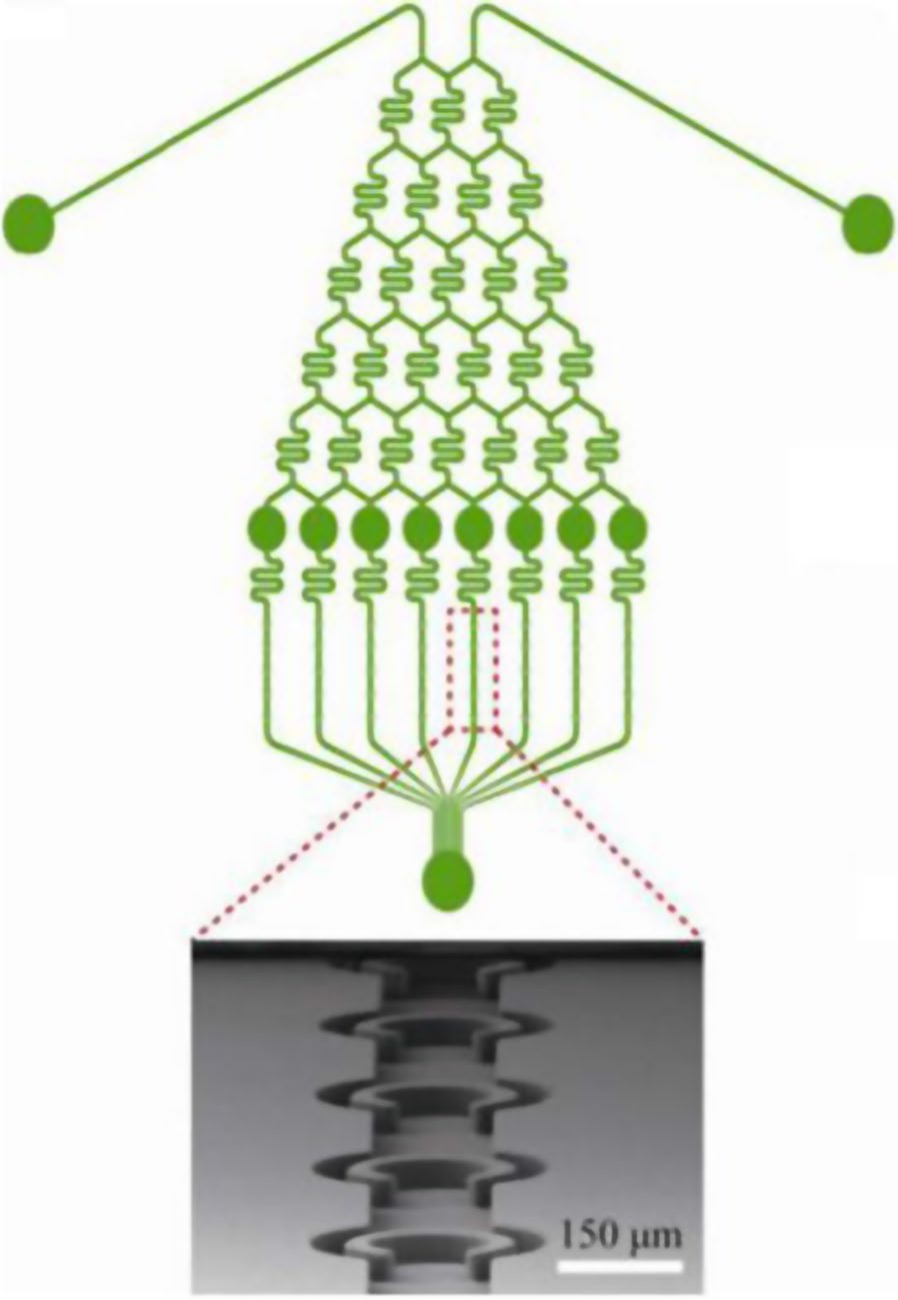
Concentration gradient generating microchannel for culture medium screening [17]

The continuous supplement of fresh medium is also feasible in microfluidic devices using mechanical trap for microalgae cell culture. The growth of *Chlamydomonas reinhardtii* in trapping chambers was studied in [18, 40], by perfusing complete TAP medium, TAP nitrogen-depleted medium and Ca_2_-depleted medium, or a medium with herbicides (methylviologen). Serially diluted sodium acetate with eight different concentrations between 0 and 10 g L^−1^ is applied in [17] for searching the optimal concentration in enhancing the growth (5.72 g L^−1^) and lipid accumulation (10.00 g L^−1^) in *Chlamydomonas reinhardtii* (Fig. 6). Multiplexed results are also obtained from five traps sharing the same concentration of acetate. In other words, 40 tests are conducted simultaneously in each device. Similarly, Zheng et al. [48] developed a microdevice to generate a copper concentration gradient, supplying eight cultivation chambers for toxicity assessments of five microalgae strains. Exposure lasted 72 h and the copper concentration ranged from 0 to 40 µmol L^−1^. Essays were performed in batch or chemostat mode. Interestingly, chlorophyll fluorescence was found to decrease with the copper concentration in *Chlorella* sp., while it increased in the case of *Phaeodactylum tricornutum*. For ecotoxicity tests, Wang et al. [39] used the surface of an air bubble formed in an aqueous solution in a microchannel to capture microalgae cells. Effects of pH variations were then studied on the captured cell by injecting NaClO or formaldehyde into the channel. Different concentrations of NaClO (30–3·10^4^ ppm) were applied to single captured *Dunaliella salina* and *Tetraselmis Chui* cells for up to 300 s. The relative intensity of chlorophyll fluorescence of the cells along the exposure duration of NaClO shows the negative impact of NaClO on photosynthetic systems. Finally, Luke et al. [22] tested the impact of pulsed 100 ppm NH_3_ on single cyanobacteria cells to mimic natural nitrogen fluctuations and observed that chlorophyll fluorescence decreased when ammonia was injected in the culture chambers.

## Comparison of microscale and bulk culture

The use of microfluidic techniques as tools to optimize operations in larger scales has been one of the main quests for researchers in both areas of microfluidics and bioengineering. However, only a small number of studies have conducted cultivation in both microfluidic and bulk scales to assess the feasibility of projecting results from microfluidic studies to bulk operations. Table 3 summarizes the culture size, the microalgae growth rate, and the productivity of the desired microalgal products in these studies.

**Table 3:** Comparison between microscale and bulk microalgae culture

Microalgae	Refs.	Culture size	Growth rate (method)	Maximum yields
*Chlamydomonas reinhardtii*	[22]	Micro: flowed droplets between 14 and 1766 pL Bulk: flasks 100 mL	2.08 day^−1a^ (count) 2.08 day^−1a^ (count)	1.1 × 10^8^ cells mL^−1^ 1.2 × 10^7^ cells mL^−1^
	[17]	Micro: mechanical trap 904 pL Bulk: flask 100 mL	0.46–4.01 day^−1a^ (F.I.) 1.30–1.85 h^−1a^ (OD_750_)	Biomass: 5.72 g L^−1^ Lipids: 10 g L^−1^ Biomass: 5 g L^−1^ Lipids: 10 g L^−1^
	[18]	Micro: mechanical trap 1 nL Bulk: –	1.85–2.08 day^−1a^ (image transparency) 0.92–1.04 day^−1a^ (OD_600_)	–
	[31]	Micro: microchamber 500 µL Bulk: flask 250 mL	* –	Lipids: 34.9 wt% Lipids: ~ 27 wt %^a^
	[35]	Micro: microchamber 500 µL Bulk: flask 100 mL	~1.0 day^−1^ (OD_800_) –	Lipids: 131.86 mg L^−1^ day^−1^ Lipids: ~ 135 mg L^−1^ day^−^
*Chlorella protothecoides*	[35]	Micro: microchamber 500 µL Bulk: flask 100 mL	~ 1.1 day^−1^ (OD_800_) –	Lipids: 166.70 mg L^−1^ day^−1^ Lipids: ~ 170 mg L^−1^ day^−1^
*Chlorella sorokiniana*	[38]	Micro: trap – Bulk: flask –	1.75 day^−1a^ (count) 1.35 day^−1a^ (OD_730_)	– –
*Chlorella vulgaris*	[22]	Micro: flowed droplets between 14 and 1766 pL Bulk: flasks 100 mL	1.39 day^−1a^ (count) 1.39 day^−1a^ (count)	4.5 × 10^8^ cells mL^−1^ 2.5 × 10^8^ cells mL^−1^
	[24]	Micro: static droplet ~ 10 nL Bulk: bioreactor 1L	0.55 to 1.52 day^−1^ (count) 1.12 day^−1^ (count)	Cell density in droplet = 20 Folds in bioreactor
	[35]	Micro: microchamber 500 µL Bulk: flask 100 mL	~1.2 day^−1^ (OD_800_) –	Lipids: 160.83 mg L^−1^ day^−1^ Lipids: ~ 150 mg L^−1^ day^−1^
*Chlorella zofingiensis*	[35]	Micro: Microchamber 500 µL Bulk: flask 100 mL	~ 1.1 day^−1^ (OD_800_) –	Lipids: 147.98 mg L^−1^ day^−1^ Lipids: ~ 140 mg L^−1^ day^−1^
*Chlorella* sp.	[30]	Micro: microchamber 240 nL Bulk: flask –	0.71 day^−1^ (count) 0.63 day^−1^ (count)	3.12 × 10^6^ cells mL^−1a^ 5.20 × 10^6^ cells mL^−1a^
*Cyclotella cryptica*	[23]	Micro: EWOD droplet ~ 70 µL Bulk: flaks 30–60 mL	0.39 day^−1a^ at R.T. (OD_660_) 0.45 day^−1a^ at 14^o^C (OD_660_)	* –
*Dunaliella tertiolecta*	[22]	Micro: flowed droplets between 14 and 1766 pL Bulk: flasks 100 mL	0.69 day^−1a^ (count) 0.69 day^−1a^ (count)	1.5 × 10^8^ cells mL^−1^ 9.0 × 10^6^ cells mL^−1^
*Haematococcus pluvialis*	[34]	Micro: microchamber 400 µL Bulk: flask 250 mL	0.25 day^−1^ –	Astaxanthin: 45.62 mg L^−1^ day^−1^ Astaxanthin: 27.63 mg L^−1^ day^−1^
*Neochloris oleoabundans*	[33]	Micro: microchamber 400 µL Bulk: flask 250 mL	2.13 day^−1^ (OD_680_) 1.34 day^−1^ (OD_680_)	*Lipids: F. I. in Micro ~ 160% In Bulk
	[35]	Micro: microchamber 500 µL Bulk: Flask 100 mL	~1.1 day^−1^ (OD_800_) –	Lipids: 144.53 mg L^−1^ day^−1^ Lipids: ~ 145 mg L^−1^ day^−1^
*Phaeodactylum tricornutum*	[30]	Micro: microchamber 240 nL Bulk: flask –	1.21 day^−1^ (count) 1.04 day^−1^ (count)	4.73 × 10^6^ cells mL^−1a^ 4.00 × 10^6^ cells mL^−1a^
*Platymonas helgolandica var. tsingtaoensis*	[30]	Micro: microchamber 240 nL Bulk: flask –	0.75 day^−1^ (count) 0.71 day^−1^ (count)	1.12 × 10^6^ cells mL^−1a^ 1.04 × 10^6^ cells mL^−1a^
*Platymonas subcordiformis*	[30]	Micro: Microchamber 240 nL Bulk: flask –	0.31 day^−1^ (count) 0.31 day^−1^ (count)	1.52 × 10^6^ cells mL^−1a^ 1.31 × 10^6^ cells mL^−1a^
*Porphyridium cruentum*	[30]	Micro: microchamber 240 nL Bulk: flask –	0.52 day^−1^ (count) 0.40 day^−1^ (count)	6.06 × 10^6^ cells mL^−1a^ 4.70 × 10^6^ cells mL^−1a^
*Scenedesmus* sp.	[35]	Micro: microchamber 500 µL Bulk: Flask 100 mL	~ 0.4 day^−1^ (OD_800_) –	Lipids: 105.42 mg L^−1^ day^−1^ Lipids: ~ 100 mg L^−1^ day^−1^
*Synechococcus elongatus*	[38]	Micro: trap – Bulk: flask –	2.28–2.92 day^−1a^ (count) 1.30–2.60 day^−1a^ (OD_730_)	– –
*Synechococcus* sp.	[38]	Micro: trap – Bulk: flask –	0.73 day^−1a^ (count) 0.62 day^−1a^ (OD_730_)	– –

–, not available

* Only optical density or fluorescence intensity is available

^a^Estimated from the reported information

### Cell proliferation

Taking advantage of the single cell resolution in microfluidic droplets, Pan et al. [25] report the extremely high cell density of *Chlamydomonas reinhardtii* (1.1 × 10^8^ cells mL^−1^), *Chlorella vulgaris* (4.5 × 10^8^ cells mL^−1^), and *Dunaliella tertiolecta* (1.5 × 10^8^ cells mL^−1^) in the 268 pL droplet compared with that in the bulk culture (100 mL flasks). For *Chlamydomonas reinhardtii* and *Dunaliella tertiolecta*, the cell density in the microfluidic droplet is ten times higher than that in the bulk culture, while it is two times higher for *Chlorella vulgaris.* Similar results are obtained by Dewan et al. [32], which shows 20 times higher cell density of *Chlorella vulgaris* in the 10 nL droplet than in the 1 L bioreactor. Interestingly, *Chlamydomonas reinhardtii* and *Chlorella* sp. grown in traps [17, 18] and microchambers [48] showed similar final cell density or biomass productivity, but much higher growth rate compared to those in flasks. Growth rates of other microalgae strains in traps [18, 22] and microchambers [42, 48] were also higher than in the flask, while those from the droplet [25] were similar with the bulk culture. The differences in the growth rate and the final cell density in different microscale cultures are likely due to the dissimilar quantification methods for cell amounts. The microscale culture conducted in mechanical traps and droplets with a thickness larger than 30 μm generally characterized the growth of microalgae cells by optical density or the autofluorescence intensity from chlorophyll, because multiple layers of cells were present in the device [17, 18, 35]. The use of autofluorescence as an indicator for cell growth can be biased by an adjustment of the photon harvesting complex to light conditions. An increase in cell density leads to the shading effect and decreases the light flux per cell, as a results, the cells increase their chlorophyll contents [74]. On comparing the growth rates obtained by cell counting in both microscale and bulk studies, one can find that the growth rates were similar in different culture scales for both droplets [25, 32] and microchambers [48]. However, the final cell density in the droplet was significantly higher than the bulk, while the microchamber had similar cell density to those in the flask [48]. Therefore, mechanical traps or chambers with relatively large volume (> 1 nL) or dimensions larger than 100 μm should be applied when using microfluidic platforms as the tool for optimizing bulk operations. A smaller culture size, such as the droplet, can result in the overestimation of cell density due to the extremely high access to lighting. Additionally, one should avoid using autofluorescence of chlorophyll as the indicator of biomass, because the reduced shading effect in the microfluidic device leads to a higher amount of pigment per cell compared with the bulk culture.

### Lipid and pigment production

In addition to cell proliferation, the lipid accumulation inside microalgae cells is also studied in both microfluidic and bulk scales. The small quantity of cells in the mechanical traps and droplets precludes the quantification of cellular contents using conventional methods such as HPLC and TLC. Therefore, in the earlier stage of microfluidic studies, quantitative comparisons between microfluidic and bulk scale culture are usually not available. Fluorescence intensities of Nile red or BODIPY labeled lipids are applied as the indicator for relative lipid amounts to search for the optimized condition for lipid accumulation. The optimized condition is then applied to the bulk culture to validate the enhanced productivity. To seek the possibility of quantitative assessments of microalgae lipids in these microsystems, Bae et al. [17] placed microalgae samples with known lipid abundance into the microfluidic trap and measured the fluorescence intensities of labeled cellular lipids. The calibration curve between fluorescence intensity and lipid abundance is established and applied to estimate the lipid abundance of *Chlamydomonas reinhardtii* cultured in the microfluidic traps. Slightly reduced lipid abundance is found in the microfluidic culture (18.07 wt%) than in the bulk culture (22.40 wt%). However, two studies report a higher fluorescence intensity from microalgal cellular lipids in microfluidic devices than in the bulk culture for *Chlamydomonas reinhardtii* [41] and *Neochloris oleoabundans* [42] and this indicates possible inaccuracy when using fluorescence intensity for the comparison of lipid abundances between microfluidic and bulk cultures. In 2014, the in situ extraction of lipids from microalgae cells in the microcolumn was developed by Lim et al. [41] and opens the door to quantifying lipid productivities in microscale. The in situ extraction leads to the validation of results from microfluidic studies and bulk operations in [45]. The lipid productivities in microfluidic and bulk culture are significantly correlated (*R*^2^ = 0.92) for the eight microalgae strains applied in their study.

However, the productivities of microalgal pigments in microscale and bulk cultures were not similar as found in [43], in which the productivity of astaxanthin in *Haematococcus pluvialis* in the microcolumn was 165% of that in a 100 mL flask. Astaxanthin is produced by *H. pluvialis* under the stress of high irradiation intensity; therefore, the reduced self-shading effect in microscale bioreactors facilitates its production. Nonetheless, the reduced self-shading effect prevents the direct projection of results for pigment production from microfluidic devices to bulk operations. Self-shading and external shading are inevitable during scale up; therefore, bulk operations are not able to match the extremely high productivities of photoprotective pigments in microfluidic devices. However, the uninterrupted and consistent lighting among each microalgae cell in the microfluidic device provides possibility for precisely determining light intensities leading to photoinhibition and photolimitation. The end/onset of these two phenomena in the bulk culture is extremely difficult to detect, because the amount of impaired cells is too small to affect the average properties of a bulk sample.

## Downstream treatments

Existing microfluidic techniques for downstream treatments for microalgal biofuel and biorefinery industry can be separated into three categories: biomass concentration, cellular contents extraction, and biomass transformation. The fabrication of downstream devices may often require the use of specific materials able to resist harsh pressure, temperature, or solvent.

### Biomass concentration

Wang and Dandy [75] built an inertial focusing microfluidic device to concentrate the cyanobacteria *Cyanobacterium Synechocystis* with hydrodynamic forces. The structure of the fluidic network passively drives the cyanobacteria laterally toward a known equilibrium position in the channel cross section. The device is composed of a filter region, an asymmetric serpentine channel, and an isolate region containing three outlets: one in the center for collecting concentrated cells and two for removing excessive medium. With a flow rate of 100 µL.min^−1^, the energy consumption of the system was estimated to be in the range of 1.3 and 8.1 kWh m^−3^ depending on the concentration factor aimed (ranging from 3 to 390). Godino et al. [76] used a similar inertial microfluidic device with three inlets and three outlets to purify microalgae from bacteria contamination and obtained purification factors up to 99.8% for the diluted microalgae sample. The concentration factors obtained by the microfluidic technique are superior compared with those obtained in larger-scale operations. At large scale, Pofleee et al. [77] previously obtained a maximum concentration factor for *Chlorella* suspensions of 1.3. Rakow and Fernald [78] obtained a concentration factor of 3 for *Spirulina* suspensions. Considering the aspect of energy efficiency, the energy consumption of microfluidic techniques could be further minimized by reducing the flow rate and multiplying the channels. However, these improvements are accompanied by high initial investment costs.

### Cellular contents extraction

Because common polymeric materials applied in rapid prototyping of microfluidic channels cannot withstand the harsh pressure, temperature, and solvents applied in conventional physical, mechanical, and chemical treatments, electroporation becomes a convenient and favorable process to weaken the cell outer compartments. Starting from 2010, microfluidic electroporation has been applied on the aqueous extraction [79], gene transfection [80, 81], and molecule delivery [82, 83] for microalgae. Owing to the closely arranged electrodes in microfluidic systems, extremely low voltage (1 V–50 V) can be used to generate an electric field larger than thousands of voltage per centimeter. The microfluidic extraction of RNA from *Karenia brevis*, which generally form cyst and are difficult to break, has more than two times higher efficiency than commercial lysis buffer as reported in [79]. Bodénès et al. [84] built a microdevice to study the in situ permeabilization of microalgae and optimize treatment parameters for lipid extraction. Chrome/gold electrodes are patterned on quartz or glass substrate with a layer of SU8 chamber to trap *Chlamydomonas reinhardtii* cells in electroporation chambers. The system enabled to observe direct penetration of propidium iodide through permeabilized membranes and evaluate the efficiency of various treatments. Results showed that pulse electric fields permeabilized membrane at a low energy consumption, but cell wall prevents the lipid leakage. Therefore, high-efficiency lipid extractions from microalgae can be facilitated by electroporation, but cannot be accomplished by electroporation alone. The lower efficiency of applying sole electroporation on the lipid extraction compared with the solvent extraction is also reported in [85]. The large-scale lipid extraction from *Chlorella vulgaris* by the continuous pulsed electric field had a throughput of 0.72 L min^−1^ and a 51% efficiency of the commercial solvent extraction. Bensalem et al. [86] studied the association of electrical treatments and mechanical stress in microsystems that affects both plasma membranes and cell wall to compare lipid recovery with solvent extraction (hexane). Observations showed that lipid extraction was correlated with cell lysis, and the combination of pretreatments weakened cells prior to solvent extraction. It is worthy to note that the extremely difficult in situ measurement of extracted lipid inside microfluidic devices can also be blamed for its supposedly low lipid extraction efficiency. The minute amount of extracted oil in microfluidic device, which leads to largely reduced fluoresence/absorbance intensity, constrains the use of dyes and conventional instrument; however, in large-scale studies, accurate and quantitative analysis can be carried out (e.g. HPLC and GC) [87].

As mentioned in the previous section, the in situ solvent extraction of lipids from *Chlamydomonas reinhardtii* in the microcolumn was developed by Lim et al. [41]. Micropillars made of PDMS are placed at the outlet of the microcolumn to retain microalgae cells in the bioreactor for the in situ extraction. The common organic solvents applied in the Bligh–Dyer method (chloroform/methanol) are not applicable in the in situ extraction because PDMS absorbs chloroform easily [88, 89]. The authors selected ethanol and isopropanol (IPA) to perform the extraction because they are more benign to PDMS, while having good abilities in extracting lipids. Two sets of bulk-scale lipid extractions were also carried out: one using the same conditions as in the microscale and the other using the Bligh–Dyer method to serve as the reference. Although ethanol and IPA resulted in lower extraction efficiencies than the Bligh–Dyer method in the bulk scale, they both had higher extraction efficiencies in the microscale and extracted up to 136% (70 wt% IPA) of total lipids compared with the Bligh–Dyer method. However, the compositions of the in situ extracted lipids had several differences than those from the Bligh–Dyer method. Due to the higher hydrophobicity of chloroform, the Bligh–Dyer method extracted more saturated lipids (C16:0, C20:0), while IPA extracted more polyunsaturated lipids (C18:3). The abundance differences of these lipids were around 5%–10% between the two methods. On the other hand, microscale and bulk-scale lipid extractions by IPA produced highly similar compositions except around 5% differences in C16:0 and C16:1. The same group extended this method to cultivate and extract lipids from eight different microalgae species on a complex microfluidic system and achieved extraction efficiencies comparable to the Bligh–Dyer method [46]. These microfluidic cultivations with integrated lipid extraction successfully demonstrated their efficiency in serving as the tool for lipid accumulation screening. When robust materials such as ceramics [90] are applied to fabricate microfluidic devices, the optimization of extraction with organic solvents can also be carried out in microscale.

### Biomass transformation

Transesterification of lipids in microreactors has been practised since 2005 [91], but almost all studies focus on the transesterification of vegetable oils and waste cooking oils. A previous review [92] summarizes the design principles, operating parameters, and catalysts for conducting transesterification in microreactors. Recently, Liu et al. [93] studied the in situ transesterification of microalgae using a microreactor. The microreactor, a 20 m-long PTFE capillary with a 0.3 mm inner diameter, was immersed in an oil bath for temperature control. Microalgae pellets were mixed with H_2_SO_4_, methanol, and chloroform and injected into the capillary for conducting transesterification. Comparison of four different microalgae species in the microreactor shows that the cell wall did not limit the efficiency of direct transesterification of fresh microalgae cells. Such process could be used for rapid fatty acid composition analysis or continuous biodiesel production directly from wet microalgal cells.

Hydrothermal liquefaction (HTL) is a downstream process combining high pressure and temperature to depolymerize the biomass into small compounds and recombine them into reactive products such as crude oil. Cheng et al. [94] have constructed a microfluidic device made of glass and silicon, able to resist temperature and pressure up to 320 °C and 12 MPa. The reaction chamber was equipped with a borosilicate glass which enabled the in situ observation of the microalgal biomass and its resulted biocrude oil. The reaction kinetics was estimated from the change of light absorbance at 675 nm (chlorophyll) and 510 (aromatic products). The results show a 1-min treatment under 320 °C and 12 MPa is sufficient to break down the cell wall, but the size of the debris is large, resulting in easily clogging. The optimized treatment duration for maximum biomass conversion and reduced debris clogging is between 2 min to 10 min.

### Future developments

#### Lipid extraction and transesterification (temperature, pressure, or solvent-resistant systems)

**Fig. 7:**
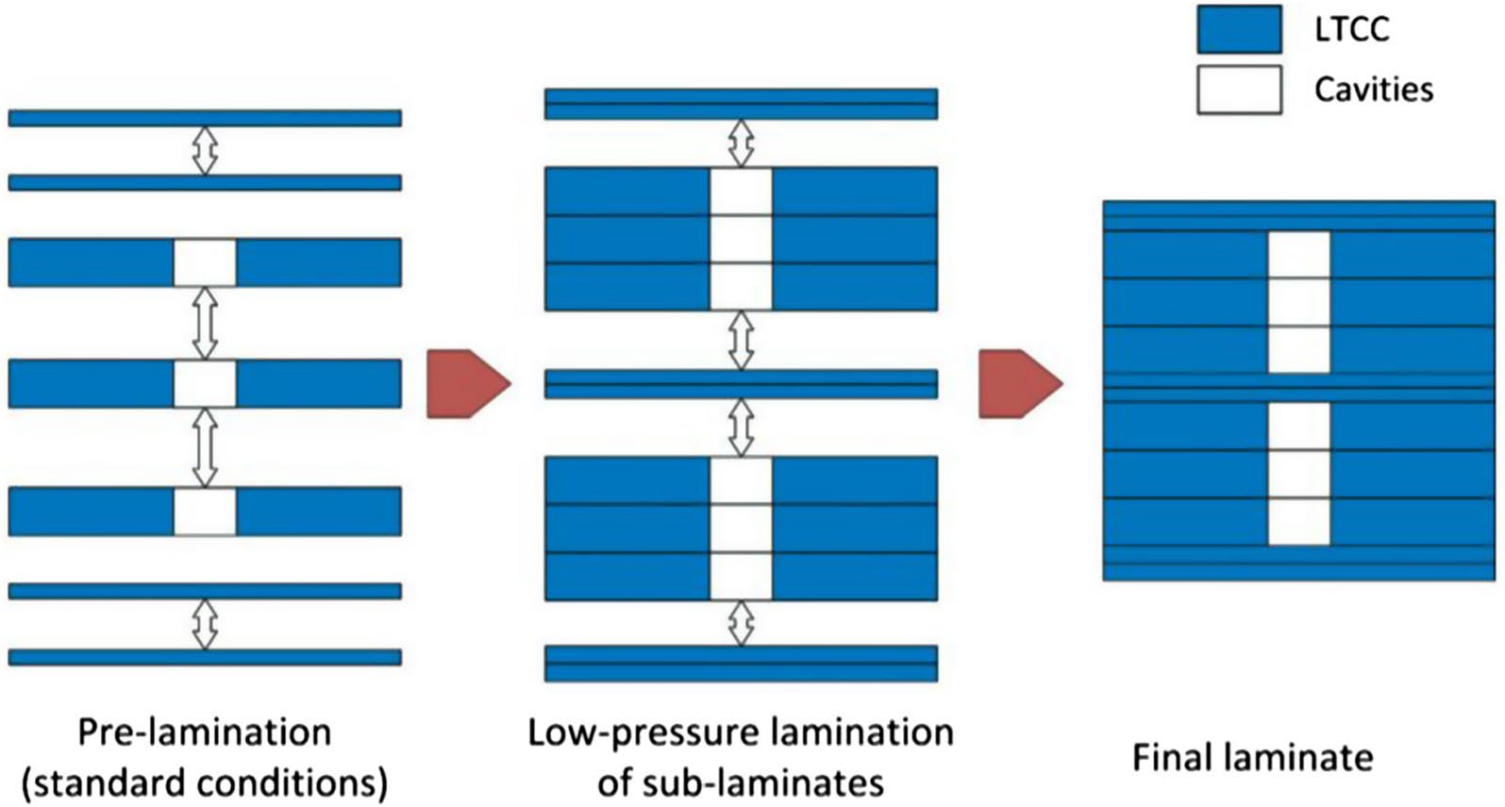
A schematic view of the multi-step lamination process [102]

**Fig. 8:**
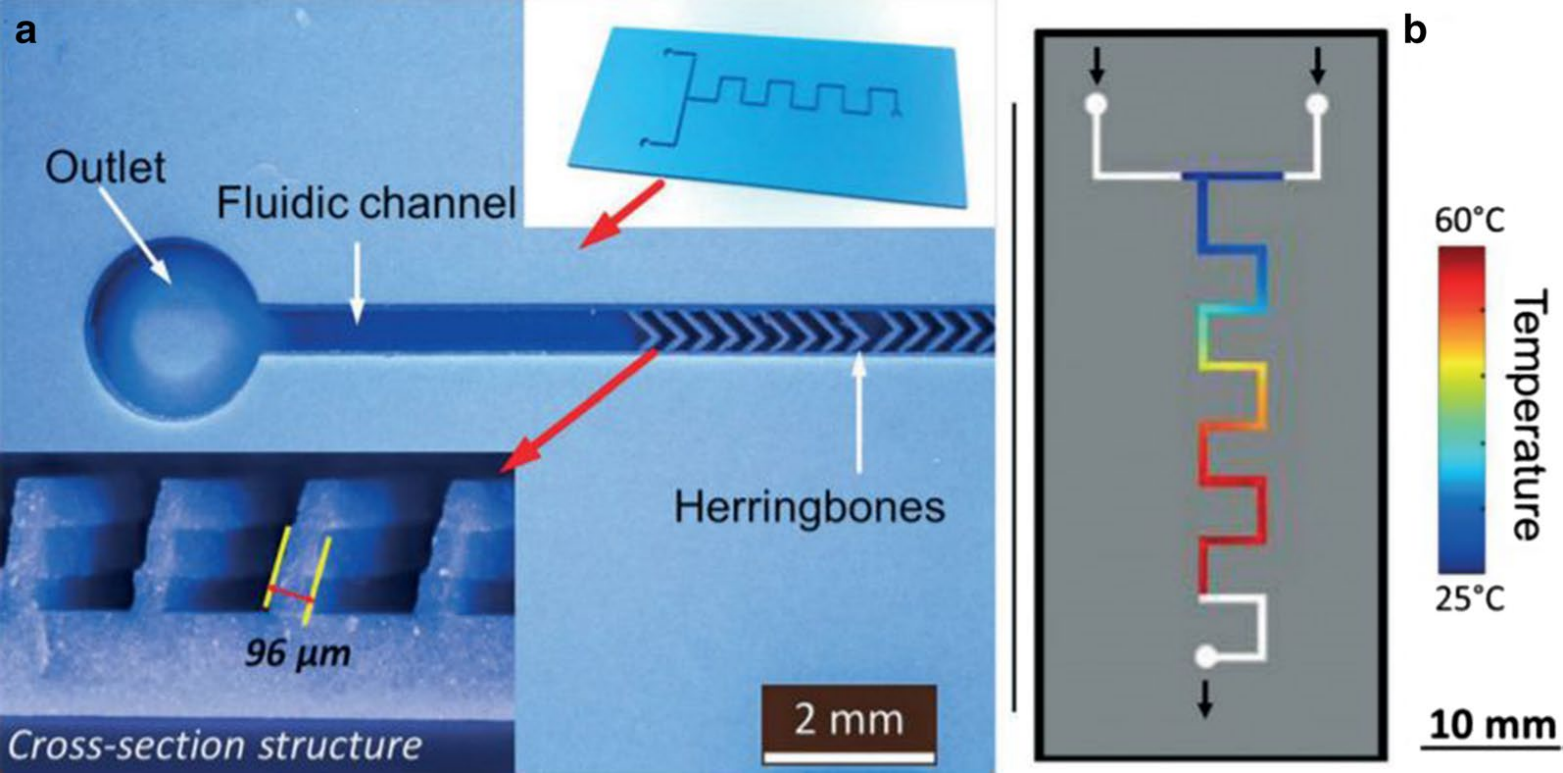
An LTCC microreactor (without a top cover) with herringbone structures for chemical mixing applications: **a** microstructural images of a fabricated microreactor containing a staggered herringbone structure in a fluidic channel; **b** infrared results of the microreactor channel mixing of sulfuric acid (7.5 mol L^−1^) and pseudoionone (1.2 mol L^−1^) at a low flow rate (0.12 m s^−1^) [102]

Compared with the screening and culture optimization of microalgae, the number of microfluidic studies for downstream treatments is extremely small. There are two main reasons limiting the progress of the research: (1) the reaction conditions of extraction and transesterification are not compatible with polymers commonly utilized for rapid molding of microfluidic devices; (2) the upscaling of microfluidic cell concentration and lysis techniques is impractical. Common polymers for rapid molding of microchannels, such as PDMS and PMMA, are not resistant to organic solvents applied in the extraction process and will react with NaOH utilized in the esterification process [95, 96]. Therefore, inert materials such as glass [94], silicon [97], or ceramics [98] have to be adapted for future studies of microfluidic extraction and transesterification of microalgal lipids. The emulsion of hydrophilic solutions and solvents, resulting in enormous contact area between two phases, has been studied in these inert microfluidic devices and provides valuable information for enhancing extraction efficiency and transesterification reaction rate. The high‐throughput (25 mL h^−1^) step emulsification of organic solvents and water for producing functional polymers was accomplished in a glass microfluidic device, which withstood the application of chloroform, toluene, and dichloromethane [99]. The water-in-diesel nanoemulsion for reducing the pollutant emission during the combustion was conducted in a ceramic microfluidic device for achieving homogeneous combustion properties [98]. Although silicon and glass have been used to fabricate microchannel extensively from 1980s, their manufacturing processes are significantly harsher comparing with the soft lithography [100] and hot embossing [101], which require relatively low temperature (60 ~ 150 °C) and mild reaction conditions. For example, the patterning of microchannel on glass requires etching with hydrogen fluoride, which is highly lethal even with a minute amount. Among inert materials, the low-temperature co-fired ceramics (LTCC) have become favorable choices in various areas of research because microfluidic structures can be straightforwardly fabricated using lamination of multiple layers of LTCC tapes, as illustrated in Fig. 7 and sintering at around 850 °C [102, 103]. LTCC-based microfluidic devices can withstand relative high temperature (~ 400 °C) [104] and harsh conditions such as strong base (NaOH) and acid (sulfuric acid) as illustrated in Fig. 8. LTCC have been widely applied in fabricating microscale components such as microsensors [90], microreactors [102], and micromixers (for emulsion) [105, 106]. A three-dimensional microfluidic device made of LTCC has been reported in [107], demonstrating the liquid–liquid partial extraction of acetone with returning extraction efficiencies around 80%. The principles and applications of LTCC on chemical process miniaturization are summarized in a recent review [108]. The results from these microscale emulsion and extraction are highly informative for performing extraction and transesterification of microalgal lipids inside microfluidic devices.

#### Techniques for studying microalgae omics

Except for lipids, microfluidic techniques can be equally useful in the research of microalgal proteins and nucleic acids. For example, the extraction and analysis of amino acids from *Dunaliella salina* using electroporation and electrophoresis is reported in [109]. The extraction efficiency of amino acids was comparable to the conventional accelerated solvent extraction method and the electrophoresis combined with laser-induced fluorescence provided a sensitivity between 3 and 9 nM. Microfluidic electroporation [110], electrophoresis [111], on-chip labeling of amino acids [112], and fluorescence detection for proteins and amino acids are all well-established techniques owing to their usefulness in medical applications. Except for the electrophoresis, on-chip liquid chromatography has also been developed for bioanalysis since 1990 and advanced greatly since [113]. The on-chip liquid chromatography has also been coupled with mass spectrometry for metabolite detections [114, 115]. Moreover, microfabricated mass analyzers and miniaturized mass spectrometers have been developed and demonstrated in numbers of applications [116]. With the introduction of these advanced technologies to the microscale microalgae research, the compositions of metabolomes and the flow of carbon/nitrogen inside the metabolome can be investigated with significantly higher throughput and lower costs in time, labor, and reagents compared with conventional analysis.

The microfluidic technologies for nucleic acid research are also well established and have proven their usefulness in various areas such as pathogen detection, rapid screening of disease markers, and genome/epigenome analysis [117]. Similar to the microfluidic protein analysis, the majority of these nucleic acid analyses are developed for medical applications and only few are applied on microalgae research, limiting the amplification of microalgal RNA on microfluidic device for the detection of toxic microalgae [118, 119] and the investigation the single cell stress response [120]. On the other hand, the number of studies of microfluidic nucleic acid techniques for bacteria is abundant and can be easily modified for the applications on microalgae. For example, the integration of sample concentration, total genome extraction and quantification for *Salmonella typhimurium* has been demonstrated in [121]. The genome DNA was extracted by on-chip electroporation with an efficiency up to 45%, which was similar to that of the commercial chemical cell lysis reagent. Although the extraction of microalgal cellular contents by the electroporation and the treatment of nucleic acids on microfluidic devices have been practised with proven efficacy, the adaptation of technologies developed in [121] for microalgal total genome may, however, require a preliminary step of cell wall degradation. Many techniques are studied in bulk scale to disrupt the cells before extraction: bead milling, ultrasonication, microwave radiation, enzymatic treatment, cell homogenizer, and high-pressure cell disruption [122]. Among these techniques, enzymatic treatment and high-pressure cell disruption may be reproduced at a microscale to have a precise control of treatment conditions (temperature, pressure, mixture homogeneity, etc.…) to ease the screening of treatment parameters and microalgae strains. Microfluidic device also facilitates the in situ visualization to directly study the effects of the above treatment conditions on cell wall, membrane, and metabolomes.

The epigenome referring to the set of chemical compounds that regulate gene expression is another important topic for understanding the metabolism of microalgae, but remains underexamined. Several studies have successfully performed epigenomic analysis such as DNA methylation and histone modification, using extremely low amount of cells in microfluidic devices [123–127], and provide valuable information for establishing microfluidic epigenomic assays for microalgae. A simple, yet high-throughput microfluidic device capable of performing multiplexed histone modification is applied to reveal the epigenomic variation between distinct brain sections in [127]. The diffusion-based microfluidic bisulfite conversion for DNA developed in [126] integrates the denaturation, sulfonation, desalting, desulfonation, and elution of DNA to effectively prevent DNA denaturation and loss due to the complex procedure. Although these epigenomic studies in microfluidic devices are currently limited to animal cells, the device design and assay principles are equally effective for studying microalgae and can be straightforwardly incorporated into existing microfluidic techniques.

#### Biofilm reactor development

Biofilm culture of microalgae appears to be a promising development path for the microalgae industry, because it has the advantages of straightforward harvesting, high mass transfer rate, high dry mass content, and reduced water consumption [128–130]. Outdoor algae biofilm production at pilot scale has been demonstrated on sandpaper rotating disk with varying productivities ranging from 0.5 to 8.4 g m^−2^ day^−1^ over half a year [131]. Polystyrene foam was used to grow *Chlorella vulgaris* as biodiesel feedstock with a fatty acid methyl ester yield of 2.59 g m^−2^ and a productivity of 0.26 g m^−2^ day^−1^ [132]. A large part of researches have been focused on finding the best adhering surface characteristics for biofilm growth such as roughness and surface energy [133, 134]. Other parameters including lighting conditions and nutrient limitation have also been studied for their effects on the metabolic status of immobilized cells in the microalgae biofilm [135, 136]. Nowack et al. developed, at microwell scale, multi-layer support for an efficient microalgae adhesion layer (porous membrane) and nutrient diffusion layer (glass fiber) [137]. Zheng et al. [138] sprayed polytetrafluoroethylene (PTFE) emulsion on glass surface to improve its wettability, which has been demonstrated to promote algae adhesion [139]. Kreis et al. [140] recently used in vivo force spectroscopy to demonstrate that *Chlamydomonas reinhardtii* show different attachment responses depending on light, indicating stronger adhesion under blue light compared to red light.

Biofilms culture in microsystem are largely studied with bacterial cells [141, 142], especially in the aspects of hydrodynamic forces and soluble chemical gradients, and these techniques can easily be adapted for microalgae culture. For example, Rusconi et al. [143] applied five straight microchannels with different widths to rapidly study the effect of shear stress on the transition of planktonic to biofilm growing state and found that 10–20 s^−1^ promotes the formation of *Pseudomonas aeruginosa* biofilm. Song et al. [144] provided new advances by correlating spatial distribution of *Pseudomonas aeruginosa* biofilm thickness with flow field distributions and chemical gradients. The responses of marine bacteria *Vibrio cyclitrophicus* toward the dynamic change of nutrients in a microchannel capable of releasing serine from side walls were studied by Yawata et al. [145] and revealed that the dissimilar abilities in forming biofilms between populations played an important role in ensuring their stable coexistence. Different from the majority of microfluidic studies for biofilm formation, which utilize image analysis to quantify the area of biofilm and the amount of bacteria in the biofilm, Kim et al. [146] used a surface acoustic wave sensor to detect bacteria biofilm growth in a microchannel by measuring the resonant frequency of the system. The sensor was made of a 400 nm-thin electrode delivering an operational frequency of approximately 400 MHz and the detection limit was approximately 166 pg of biofilm. Such quantification method can overcome the difficulties in measuring the minute amount of microalgae biomass in microfluidic culture and provide quantitative information for evaluating the feasibility of upscaling. Overall, microfluidic techniques may provide significant advances for the development of microalgae biofilm cultures, thanks to a better understanding of adhesion surface patterns, hydrodynamic forces, as well as light and nutrient gradients.

## Conclusion

The use of microfluidic systems to study microalgae has gained interest in the last decade, as evidenced by the increased number of publications on microscale technologies for microalgae screening, metabolites production, and the development of downstream processes. Great advances have been made to improve cell culture, metabolite production, and cellular composition analysis at a microfluidic scale. Downscaling the culture enables to grow single cells under optimal conditions with open-ended light, nutrient compositions, and gas transfer rate, therefore achieving higher cell density in comparison to bulk culture. This new technology has been accompanied by the development of adapted techniques for in situ growth characterization such as automated image analysis, optical density analysis, and electrical sensing. Various choices of technologies are available; the selection depends on the research objectives. Single cell analysis or study of cell population will determine the scale of the culture device and depend on whether the user wants to study an ideal case or prefers to mimic large-scale culture. Additionally, the requirement to study batch (close system) or continuous culture will determine the technology used.

Microfluidic techniques are also particularly relevant to study the application of environmental stress to trigger the accumulation of secondary metabolites such as pigments or lipids. The number of microscale culture units can be multiplied to screen a great number of conditions. Despite extensive efforts to improve the in situ quantification of these metabolites using dielectric or fluorescence characterization, further enhancements must be accomplished to facilitate the accurate estimation of microalgae composition with a minute biomass. The quantification of cellular lipids and pigments is only available when the microcolumn (~ 500 μL) is applied for microalgae culture. The development of microfluidic downstream processes is still at an early stage, because it often requires developing specific technologies or using adapted materials. Recent studies have enabled us to get a better insight into developing effective techniques for biomass concentration, biomass transformation, and metabolite extraction in microscale. Low-temperature co-firing ceramics are promising materials in building inert and resistant microchannels for the above techniques.

Finally, the study of microalgae omics and the development of biofilm reactors are two promising paths for future microfluidic studies. The microfluidic extraction and quantification of aqueous cellular contents, such as nucleic acids and proteins, are studied intensively for bacteria and these studies provide highly valuable information for obtaining omics data of microalgae. The precise control of surface properties, hydrodynamic forces, and environmental factors in microfluidic device significantly facilitates the study of their effects on microalgal biofilm formation. With the success of these research topics, increased microalgal product values and reduced production costs (for culturing and downstream treatments) can be largely achieved with promises of profitable biofuel and biorefinery industry based on microalgae.

## Abbreviations

EMCCD: electron-multiplying charged coupled device
PDMS: polydimethylsiloxane
PMMA: poly(methyl methacrylate)
BODIPY: boron-dipyrromethene
TAP: tris–acetate–phosphate medium
BBM: bold basal medium
EWOD: electrowetting on dielectric
LED: light-emitting diode
PMT: photomultiplier tube
LP: long pass filter
CCD: charge-coupled device
CMOS: complementary metal–oxide–semiconductor
FEP: fluorinated ethylene propylene
OLEDS: organic light-emitting diodes
OPD: organic photodetectors
FDA: fluorescein diacetate
RPS: resistance pulse sensor
DEP: dielectrophoresis
iDEP: insulator-based dielectrophoresis
ROS: reactive oxygen species
HPLC: high-performance liquid chromatography
TLC: thin layer chromatography
SU8: epoxy-based negative photoresist
IPA: isopropanol
PTFE: polytetrafluoroethylene
HTL: hydrothermal liquefaction
FA: fatty acids
LTCC: low-temperature co-fired ceramics
